# Insights on FXR selective modulation. Speculation on bile acid chemical space in the discovery of potent and selective agonists

**DOI:** 10.1038/srep19008

**Published:** 2016-01-07

**Authors:** Valentina Sepe, Carmen Festa, Barbara Renga, Adriana Carino, Sabrina Cipriani, Claudia Finamore, Dario Masullo, Federica del Gaudio, Maria Chiara Monti, Stefano Fiorucci, Angela Zampella

**Affiliations:** 1 Department of Pharmacy, University of Naples “Federico II”, Via D. Montesano, 49, 80131 Naples, Italy; 2 Department of Surgery and Biomedical Sciences, Nuova Facoltà di Medicina, P.zza L. Severi 1, 06132 Perugia, Italy; 3 PhD Program in Drug Discovery and Development, University of Salerno, Via Giovanni Paolo II 132, 84084 Fisciano (Salerno), Italy; 4 Department of Pharmacy, University of Salerno, Via Giovanni Paolo II, 132, 84084 Fisciano (Salerno), Italy

## Abstract

Bile acids are the endogenous modulators of the nuclear receptor FXR and the membrane receptor GPBAR1. FXR represents a promising pharmacological target for the treatment of cholestatic liver disorders. Currently available semisynthetic bile acid derivatives cover the same chemical space of bile acids and therefore they are poorly selective toward BA receptors, increasing patient risk for adverse side effects. In this report, we have investigated around the structure of CDCA describing the synthesis and the *in vitro* and *in vivo* pharmacological characterization of a novel family of compounds modified on the steroidal tetracyclic core and on the side chain. Pharmacological characterization resulted in the identification of several potent and selective FXR agonists. These novel agents might add utility in the treatment of cholestatic disorders by potentially mitigating side effects linked to unwanted activation of GPBAR1.

Farnesoid X receptor (FXR) is one of the 48 ligand-activated nuclear transcription factor proteins. Mostly expressed in hepatocytes, biliary epithelium, small bowel enterocytes, renal tubular cells and adrenal glands, FXR senses the intracellular presence of bile acids (BAs) by inducing changes in gene expression^1^,^2^. The results of the consequent activated multiple mechanisms include promotion of bile acid export from liver, down-regulation of bile acid import and also attenuation of *de-novo* bile acid synthesis^3^. Thus, FXR has been identified as an appealing target in the treatment of cholestasis and liver steatosis^4^,^5^,^6^. In addition to its canonical role in regulating bile acid homeostasis, FXR plays a crucial beneficial role in hepatic triglyceride (TG) homeostasis, as well as in glucose metabolism. FXR lowers hepatic TG content and serum TG levels and improves insulin resistance and hyperglycemia. Therefore, FXR agonists are promising for the treatment of non-alcoholic fatty liver disease (NAFLD), dyslipidemia and type 2 diabetes^7^,^8^,^9^,^10^.

Among endogenous occurring BAs, chenodeoxycholic acid (CDCA, **1** in Fig. 1) is the most potent natural FXR ligand (EC_50_ ~ 10 μM), whereas deoxycholic acid and lithocholic acid are weaker natural ligands^11^.

In addition, secondary BAs activate the membrane G-protein-coupled receptor GPBAR1 (also known as M-BAR, TGR5 or BG37)^12^, and the exogenous dual control over the two receptors represents an attractive strategy for the treatment of non-alcoholic steatohepatitis (NASH) and type 2 diabetes^13^,^14^,^15^.

On the other hands, GPBAR1 has been recently identified as the physiological mediator of pruritus^16^, a common symptom observed in cholestasis and the severity of this side effect limits the pharmacological utility of dual agonists in the treatment of primary biliary cirrhosis (PBC) and related cholestatic disorders. In this context, the discovery of highly selective FXR agonists, devoid of GPBAR1 agonism, represents a promising approach in the identification of new pharmacological protocols for PBC, an orphan disease for which therapeutic options are limited and poorly effective.

Indeed, in the last ten years bile acid scaffold has been subjected to intense medicinal chemistry modifications, producing several steroidal derivatives modified on the side chain in the length and in the nature of the end-group and on the tetracyclic core. Since steroidal ligands cover the same chemical space of BAs, they are intrinsically promiscuous toward FXR and GPBAR1 and, with few exception, this kind of speculation mainly afforded dual modulators^17^,^18^. Indeed, the removal or isomerization of the hydroxyl group at C-3 on LCA or its tauro-conjugated form (TLCA, **2** in Fig. 1), the most potent GPBAR1 agonist among endogenous BAs^19^, is detrimental in term of GPBAR1 agonism, and this observation was recently translated on CDCA scaffold demonstrating that 3-deoxy-5β-cholane derivatives are selective FXR ligands^17^. Independently from the functional group at C-24 on the side chain, derivatives **3**–**5** (Fig. 1) were demonstrated selective FXR agonists with 7α-hydroxy-5β-cholan-24-sulfate (**5**) transactivating FXR with an EC_50_ of ~9 μM. Even if less potent in transactivation assay than 6-ethylchenodeoxycholic acid, 6-ECDCA/OCA (**6**), the most potent steroidal FXR agonist generated so far, the potency of compound **5** in inducing the expression of OSTα, a FXR target gene in the liver, is comparable to that of 6-ECDCA. However 6-ECDCA is also a GPBAR1 activator^20^,^21^, and administration of PBC patients has led to exacerbation of itching causing drug discontinuation in 40% of patients^22^.

With this background in mind, we decided to proceed in the modification of the hydroxyl group at C-3 on 6-ethylcholane scaffold generating a library of 6-ethylcholane derivatives that are divided in two subsets (Fig. 2): subset A includes the 3-deoxy-6-ethyl derivatives, and subset B includes the 3β-hydroxyl derivatives.

As shown in the Fig. 2, in each subset, our investigation was also expanded on the side chain-end group and on the stereochemical arrangement of 6-ethyl and 7-hydroxyl substituents on ring B affording to the discovery of several derivatives as potent and selective FXR agonists.

## Results

### Modification on 6-ethylchenodeoxycholane scaffold in the preparation of subset A derivatives (Fig. 3)

Methyl ester formation and acetylation at C-3 hydroxyl group on 7-KLCA furnished intermediate **22** in 84% yield over two steps. Aldolic addition to a silyl enol ether intermediate generated **23**. Hydrogenation at the exocyclic double bond (H_2_ on Pd(OH)_2_) afforded exclusively the 6β-ethyl group in **24** (80% yield over three steps) as demonstrated by dipolar couplings Me-26 (δ 0.83)/Me-19 (δ 1.22) and H-8 (δ 2.56)/H-25 (δ 1.83) in Noesy spectrum. First, compound **24** was treated with MeONa in methanol to effect de-acetylation at C-3 and inversion at C-6 and then with tosyl chloride to afford **25** in quantitative yield over two steps. Elimination by LiBr/Li_2_CO_3_ treatment and hydrogenation of the unsaturated-ring A transient intermediate furnished the key derivative **26**. LiBH_4_ treatment produced the concomitant reduction at C-24 methyl ester and at C-7 carbonyl group furnishing **7** in high chemical yield and a small aliquot of the corresponding epimer at C-7 (**8**), efficiently separated by HPLC.

Chemoselective sulfation at C-24 hydroxyl group on a small aliquot of **7** gave the corresponding sulfate derivative **9**. Intermediate **26** was also used as starting material for the preparation of compounds **10** and **11**. Alkaline hydrolysis of the methyl ester followed by LiBH_4_ treatment furnished **10** in high chemical yield with about 10% of 6α-ethyl-7β-hydroxy-5β-cholan-24-oic acid (**11**) that was isolated by HPLC. Amidation with taurine on the major 6α-ethyl-7α-hydroxy-5β-cholan-24-oic acid (**10**) and C18 silica gel column/HPLC purification provided the corresponding tauro-conjugated **12**.

As previously demonstrated, stereochemical arrangement of the substituents at C-6 and C-7 on ring B profoundly affects bile acid receptor selectivity^17^. Invariably the presence of the 6α-ethyl and the 7α-hydroxyl groups produces potent agonists with dual FXR/GPBAR1 activity whereas the 6-epimers (6β-ethyl derivatives) are selective GPBAR1 agonists. To access to 3-deoxy-6β-ethylchenodeoxycholane derivatives, compound **24** was treated with MeONa in MeOH for 2 h reaction time affording de-acetylation at C-3 without epimerization at C-6. Tosylation on the crude reaction product furnished **27** that was subjected to the same operative condition described for compound **26**, to obtain **28** in 94% yield over two steps. Treatment of **28** with NaBH_4_ in methanol followed by LiBH_4_ reduction on the crude reaction product to secure the complete reduction of the methyl ester on the side chain afforded a mixture whose HPLC purification gave pure **13** in a 54% yield respect to the corresponding C7 epimer, compound **14**.

### Modification on 6-ethylchenodeoxycholane scaffold in the preparation of subset B derivatives (Fig. 4)

As previously demonstrated, modification at C-3 impacts on BAs selectivity and activity towards FXR and GPBAR1. Isomerization of the 3*R*-hydroxyl group on LCA robustly attenuates the agonistic activity on GPBAR1 with iso-LCA less potent respect to the cognate LCA. On the other hand, isochenodeoxycholic acid (iso-CDCA) is more potent than CDCA in transactivating FXR and notably has only very weak activity towards GPBAR1^17^.

With this background in mind, our speculation on 6-ethylchenodeoxycholane scaffold was expanded to the preparation of 3β-hydroxyl derivatives.

As depicted in Fig. 4, in a convergent protocol, inversion at C-3 on **27** with potassium acetate in DMF/H_2_O followed by treatment with MeONa/MeOH gave **29** (74% over two steps) that was used as starting material in the synthesis of derivatives **15**–**19**. In detail NaOH hydrolysis followed by LiBH_4_ treatment gave **17** and small amounts (about 10%) of the corresponding 7β-hydroxyl derivative **18**, efficiently separated by HPLC. Tauro-conjugation on a small aliquot of **17** proceeded smoothly with the formation of **19** in good chemical yield. In a parallel protocol, intermediated **29** was subjected to LiBH_4_ treatment affording alcohol **15** in presence of a small amount of 6α-ethyl-3β,7β-dihydroxy-5β-cholan-24-ol (**16**).

**Figure 3:**
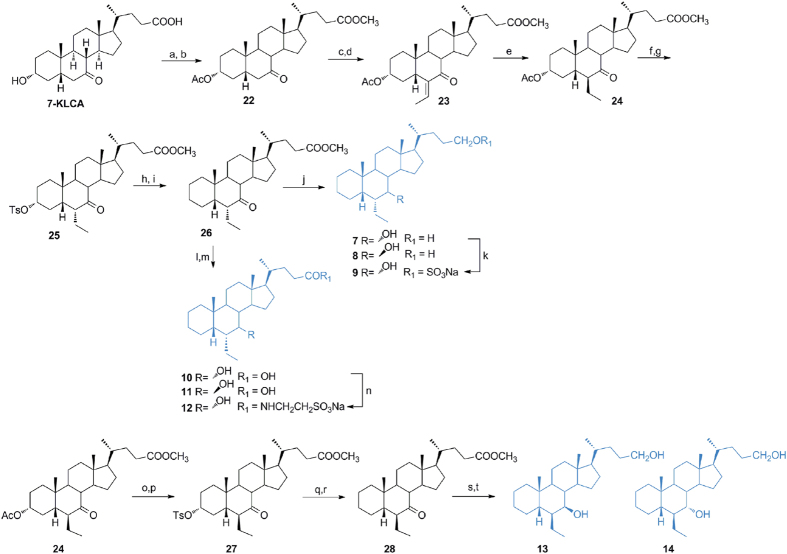
Preparation of 3-deoxy-6-ethylchenocholane derivatives (subset A). *Reagents and conditions*: (**a**) *p*-TsOH, MeOH dry; (**b**) acetic anhydride, pyridine, 84% yield over two steps; (**c**) DIPA, *n*-BuLi, TMSCl, TEA dry, THF dry −78 °C; (**d**) acetaldehyde, BF_3_(OEt)_2_, CH_2_Cl_2_, −60 °C, 80% over two steps; (**e**) H_2_, Pd(OH)_2_, THF/MeOH 1:1, quantitative yield; (**f**) MeONa, MeOH; g) p-TsCl, pyridine, quantitative yield over two steps; (**h**) LiBr, Li_2_CO_3_, DMF, reflux, (**i**) H_2_, Pd(OH)_2_, THF/MeOH 1:1, room temperature, 88% over two steps; (**j**) LiBH_4_, MeOH dry, THF, 0 °C, 77%; (**k**) Et_3_N.SO_3_, DMF, 95 °C; (**l**) NaOH, MeOH:H_2_O 1:1 v/v, 98%; (**m**) LiBH_4_, MeOH dry, THF, 0 °C, 83%; (**n**) DMT-MM, Et_3_N, taurine, DMF dry; (**o**) MeONa, MeOH; (**p**) *p*-TsCl, pyridine, quantitative yield over two steps; (**q**) LiBr, Li_2_CO_3_, DMF, reflux; (**r**) H_2_, Pd(OH)_2_, THF/MeOH 1:1, room temperature, 94% over two steps; (**s**) NaBH_4_, MeOH; (**t**) LiBH_4_, MeOH dry, THF, 0 °C, 78% over two steps.

**Figure 4:**
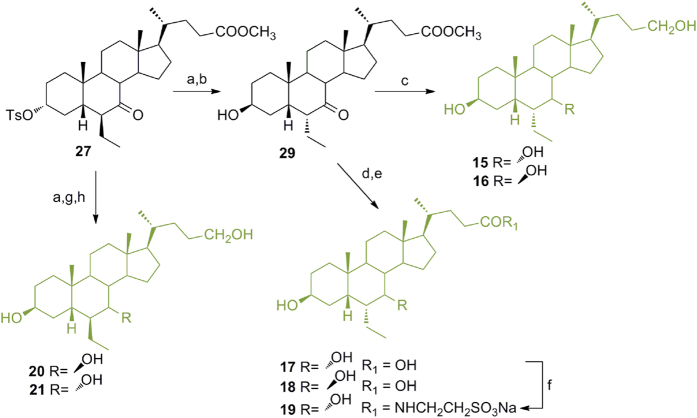
Preparation of 3β-hydroxy-6-ethylchenocholane derivatives (subset B). *Reagents and conditions*: (**a**) CH_3_COOK, DMF:H_2_O 5:1 v/v; (**b**) NaOMe, MeOH, 74% over two steps; (**c**) LiBH_4_, MeOH dry, THF, 0 °C, 58%; (**d**) NaOH, MeOH:H_2_O 1:1 v/v; (**e**) LiBH_4_, MeOH dry, THF, 0 °C, 74% over two steps; (**f**) DMT-MM, Et_3_N, taurine, DMF dry; (**g**) NaBH_4_, MeOH; (**h**) LiBH_4_, MeOH dry, THF, 0 °C, 74% over three steps.

Compound **27** was also used as starting material for the preparation of compounds **20**–**21** (Fig. 4). Inversion at C-3 followed by reduction at C-7 and C-24 with NaBH_4_/LiBH_4_ treatment as described for the corresponding 3-deoxy derivatives (Fig. 3) furnished **20** and **21** as a mixture, then separated by HPLC.

### Preparation of the reference compound, 6-ECDCA (6) (Fig. 5)

Methyl ester **24** was also converted in 6-ECDCA (**6**), used as reference compound in the pharmacological evaluation of our library of 6-ethylcholane derivatives. Basic treatment (NaOH, MeOH/H_2_O) on **24** proceeded in a straightforward manner affording the concomitant hydrolysis on side chain methyl ester, de-acetylation at C-3 and inversion at C-6 ethyl group. LiBH_4_ reduction of the C7-carbonyl group on intermediate **30** furnished 6-ECDCA (**6**) in 69% over two steps.

### *In vitro* pharmacological evaluation

**Figure 6:**
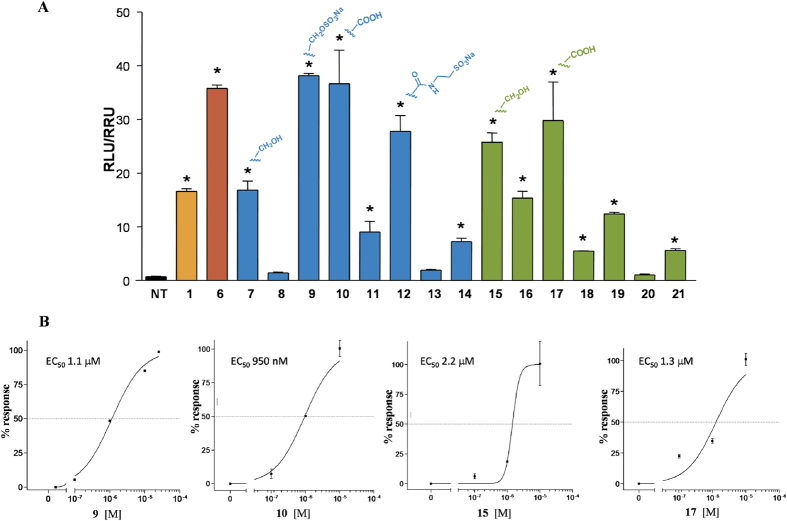
Transactivation assays on FXR. (**A**) HepG2 cells were transfected with pSG5-FXR, pSG5-RXR, pCMV-βgal, and p(hsp27)TKLUC vectors. Cells were stimulated with compounds **7**–**21** (10 μM). CDCA (**1**, 10 μM) and 6-ECDCA (**6**, 1 μM) were used as a positive control. Results are expressed as mean ± standard error; *p < 0.05 *versus* not treated cells (NT); (**B**) Concentration-response curve of **9**, **10**, **15** and **17** on FXR in a luciferase reporter assay using HepG2 cells transfected with FXR. Twenty-four hour post transfection cells were stimulated with increasing concentrations of each agent: range from 100 nM to 10 μM.

Derivatives **7**–**21** were tested for FXR and GPBAR1 activity, in a luciferase reporter assay with HepG2 and HEK-293T cells transfected with FXR and GPBAR1, respectively (Figs 6,[Fig f7], 8 and 9). As reported in Fig. 6A, all 6α/7α derivatives (compounds **7**, **9**, **10** in subset A and **15**, **17** in subset B) are potent agonists of FXR. Within 6α/7α stereochemical arrangement, the presence of a negative charge on the side chain favors the 3-deoxy derivatives with the carboxylic acid **10** and the sulfate derivative **9** potent FXR activators whereas the alcoholic function at C-24 improves FXR activity of the corresponding 3β-hydroxyl derivatives (compare **10** vs **17** and **15** vs **7**). Of interest are also the cellular assay results on compounds with one of the two substituents on ring B in a β-configuration (C6α/C7β or C6β/C7α) revealing a remarkable decrease in FXR activity when compared to the corresponding 6α/7α substituted derivatives. In detail, one again, the activity is related to the functional group on the side chain and to the presence/absence of the hydroxyl group at C-3. First derivative **8**, a C3-deoxy/C24-alcohol with the C6α/C7β stereochemical arrangement is completely inactive towards FXR whereas the corresponding carboxylic acid **11**, even if less potent than CDCA (**1**), retains a certain activity. Opposite is the behavior of the corresponding 3β-hydroxyl derivatives where the presence of the alcoholic function at C-24 on the side chain improve FXR activity (compare **16** *vs* **18**).

Finally, the complete loss of activity for compounds **13** and **20** points the attention on the pharmacophoric role of the C6/C7 stereochemical arrangement with the 6β/7β relationship detrimental in FXR binding.

**Figure 7 f7:**
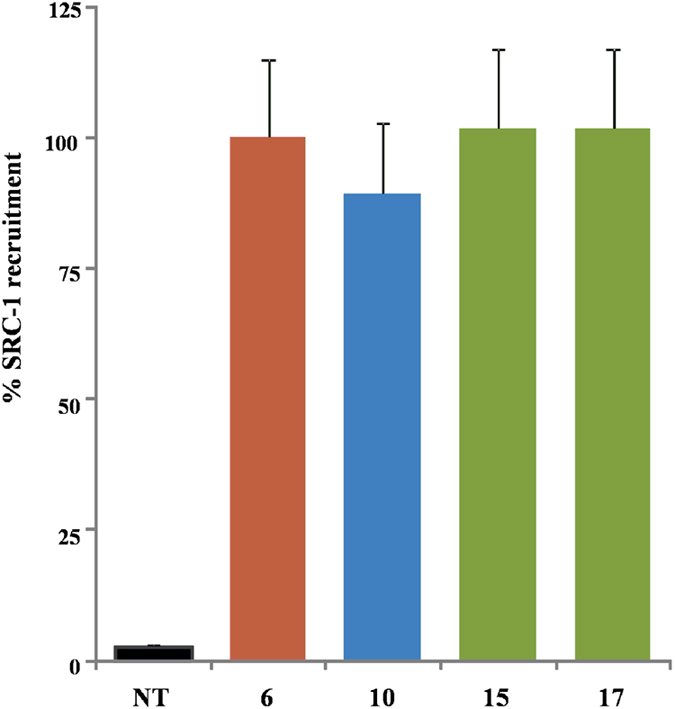
Coactivator recruitment assay measuring a direct interaction of FXR with SRC-1. Anti-GST-coated acceptor beads captured GST-fusion FXR-LBD and the biotinylated-SRC-1 peptide was captured by the streptavidin donor beads. FXR-LBD recruited SRC-1 in the presence of ligand at 2 μM and, upon illumination at 680 nm, chemical energy is transferred from donor to acceptor beads across the complex streptavidin-donor/Src-1-biotin/ligand/GSTFXR-LBD/anti-GST-acceptor and a signal is produced. Results are expressed as percentage of the effect of **6** arbitrarily settled as 100%. NT is referred to the experiment carried out in absence of ligand. Results are expressed as mean ± standard error.

The relative potency of selected members of this novel family was then investigated by a detailed measurement of concentration-response curve of the 3-deoxy sulfate derivative **9**, the corresponding carboxylic acid **10**, and the 3β-hydroxyl alcohol **15** and carboxylic acid derivative **17**, all sharing the 6α/7α configuration, on FXR transactivation. As illustrated in Fig. 6B, compounds **9**, **10**, **15** and **17** transactivate FXR with an EC_50_ of 1.1 μM, 950 nM, 2.2 μM and 1.3 μM, respectively, with compound **10** showing a comparable potency with the reference compound, 6-ECDCA, EC_50_ = 500 nM in the same assay (Supporting Information, Figure S1). Of interest, the ability in transactivating FXR is also maintained by the corresponding tauro-conjugated **12** (Fig. 6A, compare **10** *vs* **12**), thus highlighting the therapeutical potential of 6α-ethyl-7α-hydroxy-5β-cholan-24-oic acid (**10**) in human FXR mediated diseases. On the contrary, tauro-conjugation on carboxylic acid **17** produces a considerable decrease in FXR transactivation activity (compare **19** vs **17** in Fig. 6A), thus first demonstrating that a longer negative charged side chain is detrimental in FXR binding when a 3β-hydroxyl group is present on the steroidal scaffold and second, reducing the pharmacological impact of compound **17**. Finally the ability of the more active compounds in transactivating FXR was measured as recruitment assay of the coactivator SRC-1, using Alphascreen technology. In this assay, the ligand induces the recruitment of the coactivator to the LBD of hFXR. 6-ECDCA (**6**) was used as positive control and reference molecule setting its effect as 100% (Fig. 7). All tested derivatives, 6α-ethyl-7α-hydroxy-5β-cholan-24-oic acid (**10**), 6α-ethyl-3β,7α-dihydroxy-5β-cholan-24-ol (**15**) and the corresponding carboxylic acid, 6α-ethyl-3β,7α-dihydroxy-5β-cholan-24-oic acid (**17**) showed a very potent activity in the recruitment of SRC-1 co-activator and high affinity to FXR, almost comparable to that measured for **6**, thus confirming the transactivation results.

**Figure 8:**
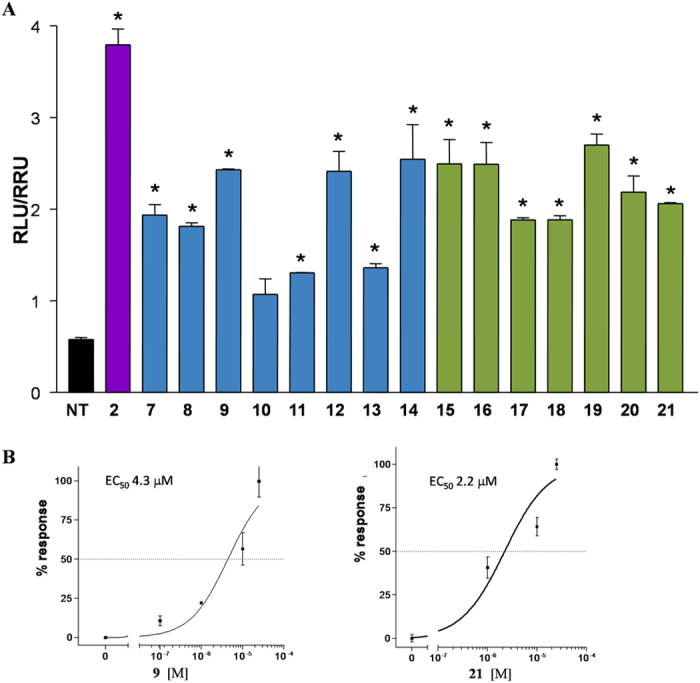
Agonism on GPBAR1 by transactivation assay. (**A**) HEK-293T cells were co-transfected with GPBAR1 and a reporter gene containing a cAMP responsive element in front of the luciferase gene. Twenty-four hour post transfection cells were stimulated with **7–21** (10 μM). Luciferase activity served as a measure of the rise in intracellular cAMP following activation of GPBAR1. TLCA (**2**, 10 μM) was used as a positive control. Results are expressed as mean ± standard error. *p < 0.05 *versus* not treated cells (NT); (**B**) Concentration-response curve of **9** and **21** on GPBAR1 in HEK-293T cells co-transfected with GPBAR1 and a reporter gene containing a cAMP responsive element in front of the luciferase gene (CRE). Twenty-four hour post transfection cells were stimulated with increasing concentrations of each agent: range from 100 nM to 50 μM. Results are expressed as mean ± standard error.

Results of transactivations of CREB-responsive elements in HEK-293T, transiently transfected with the membrane bile acid receptor GPBAR1 (Fig. 8A), revealed that the strategy of elimination or inversion at C-3 hydroxyl group could be instrumental in shifting the selectivity of 6-ethylcholane derivatives toward FXR.

Indeed, independently by the functional group on the side chain, the stereochemical arrangement of C6/C7 substituents and the presence/absence of the hydroxyl group at C-3, all derivatives generated in this study are weak agonists, substantially less potent than TLCA (**2**) in modulating GPBAR1. By way of example, the concentration-response curve on compounds **9** and **21** (Fig. 8B) resulted in EC_50_ values of 4.3 and 2.2 μM, substantially lesser than the corresponding value for TLCA (EC_50_ = 0.29 μM)^19^. As a consequent, compound **9** should be considered a preferential FXR modulator with a residual activity on GPBAR1 whereas compound **21**, less potent then CDCA in transactivating FXR (Fig. 6A), is a weak but preferential GPBAR1 activator.

**Figure 9:**
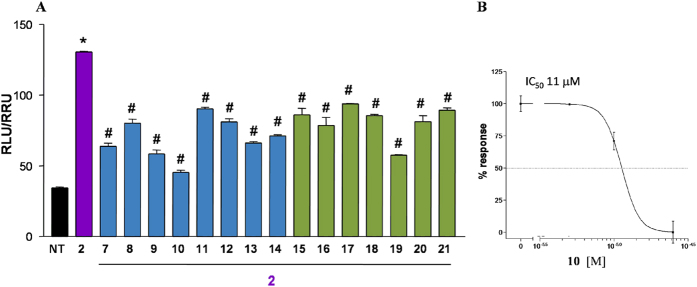
Antagonism on GPBAR1 by transactivation assay. (**A**) HEK-293T cells were transfected as described in Fig. 8. Twenty-four hour post transfection cells were stimulated with 10 μM TLCA (**2**) alone or in combination with 25 μM compounds **7–21**. ^#^p < 0.05 *versus* TLCA stimulated cells; (**B**) Concentration-response curve of **10** on GPBAR1 in combination with TLCA (**2**, 10 μM) and with increasing concentrations of **10**: range from 5 to 25 μM. Results are expressed as mean ± standard error.

Moreover, 6α-ethyl-7α-hydroxy-5β-cholan-24-oic acid (**10**), one of the most potent derivative in transactivating FXR is inactive towards GPBAR1 in agonistic mode (Fig. 8A). Of interest, when administered in presence of 10 μM TLCA (**2**), compound **10** showed inhibitory activity against GPBAR1 transactivation induced by TLCA (Fig. 9A). The above result was also confirmed by a concentration-response curve in HEK-293T transiently transfected with GPBAR1, revealing for **10** an IC_50_ value of 11 μM in antagonizing the effect of TLCA (Fig. 9B). To the best of our knowledge, this result represents the first report of a 6-ethylcholanoic derivative endowed by potent FXR agonism and able to antagonize GPBAR1. This discovery opens the way to a new and promising field of research. First, the speculation of the *in vivo* effects of this compound could result in the identification of new therapeutical approach to FXR mediated liver disorders in which the concomitant activation of GPBAR1 is associated to severe side effects. Second, compound **10** represents a novel tool compound, useful component of today’s research arsenal of BA derivatives, in dissecting and in shedding light on the complex biological pathways under GPBAR1 control.[Fig f10]

### *In vivo* pharmacological characterization (Figs. 10,11)

**Figure 10 f10:**
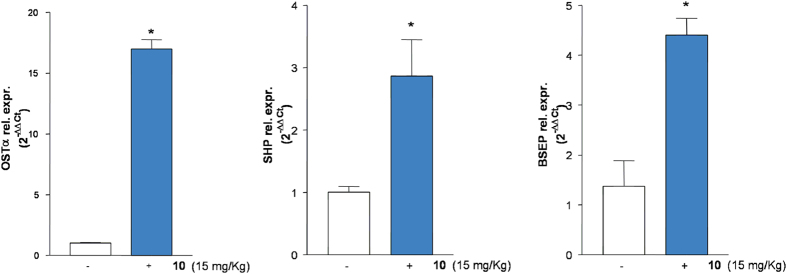
*In vivo* effect of 10 on FXR target genes. C57BL6 mice were administered with **10** (15 mg/Kg) for 6 h. After treatment liver were removed and the relative mRNA expression of OSTα, SHP and BSEP was assayed by quantitative Real-Time PCR. *p < 0.05 versus control mice.

C57BL6 mice were administered with compound **10** (15 mg/kg, ip). At 6 h post-treatment, livers and blood were collected. As shown in Fig. 10, compound **10** significantly up-regulated the relative mRNA expression of canonical FXR molecular targets such as OSTα, SHP and BSEP in the liver exposed 6 h to **10** (*p < 0.05 versus control mice).

**Figure 11:**
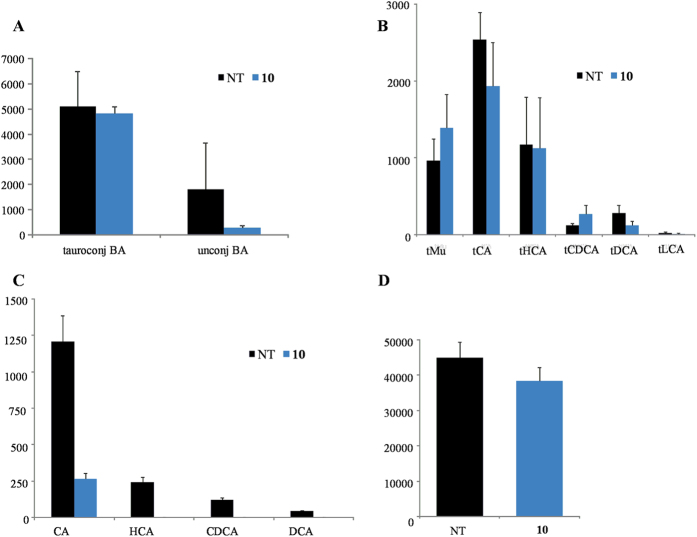
Blood concentrations of (A) total tauro-conjugated and total unconjugated bile acids; (**B**) individual conjugated and (**C**) unconjugated bile acids; (**D**) 7α-hydroxy-4-cholesten-3-one before and after *in vivo* administration of **10** at 15 mg/Kg.

Analysis of plasmatic concentrations of unconjugated and tauro-conjugated bile acids demonstrated that *in vivo* administration of **10** significantly reduced total unconjugated bile acids while the quote of tauro-conjugated tMu, tCA, tHCA, tCDCA, tDCA and tLCA was unaffected (Fig. 11, *p < 0.05 versus control mice). Compound **10** was only found in the conjugated form with taurine (data not shown).

## Discussion and Conclusion

In addition to their role in lipid absorption, and similarly to other cholesterol metabolites, bile acids are signaling molecules.

Each bile acid interacts with more than one receptor, however, FXR and GPBAR1 are differentially activated by CDCA>DCA >LCA>CA and LCA>DCA>CDCA>CA, respectively. In addition to endogenous ligands, several semisynthetic bile acids and non-steroidal FXR ligands are currently under evaluation in the treatment of metabolic (NASH) and cholestatic liver diseases (PBC).

Obeticholic acid (OCA, or 6-ECDCA (**6**), or INT-747), a semi-synthetic dual FXR and GPBAR1 ligand and a derivative of CDCA, has been recently evaluated in the multicenter, double-blind, randomized NASH (FLINT) trial^23^. Although 6-ECDCA/OCA significantly improved the primary histological outcome (NAFLD activity score) and fibrosis score compared with placebo, NASH resolution occurred in only 22% of patients after 72 weeks (p = 0.08 vs. placebo). In addition, a 5% decrease in HDL-C levels coupled with a 16% increase in LDL-C was observed with OCA as compared to placebo and the impact of these changes on long-term cardiovascular risk in NASH is unknown. One major limitation of the use of OCA is the high incidence of pruritus, occurring in up to 80% patients with primary biliary cirrhosis receiving OCA and primarily responsible for high rate of drug discontinuation in 40% of patients^23^,^24^. Indeed, OCA is a dual FXR/GPBAR1 agonist^20^,^21^, and GPBAR1 has recently been demonstrated a physiological mediator of itching^15^, indicating that highly selective FXR agonists might have utility in the treatment of PBC. In this context, we have modified BA scaffold generating a library of 6-ethylcholane derivatives having variable and peculiar activity toward FXR and GPBAR1.

From a chemical perspective of structure-activity relationship, our investigation has been focused primarily on three areas: (1) the modification at C-3 hydroxyl group (elimination/inversion) on the 6-ethylcholane scaffold; (2) changes to the end group on side chain; and (3) the stereochemical orientation of C6/C7 substituents on ring B. The first consideration to be drawn is that all 6-ethylcholane derivatives prepared in this study are at least weak GPBAR1 agonists (Fig. 8), thus affirming the key pharmacophoric role of the 3α-hydroxyl group for GPBAR1 activation by BA scaffold. Second, as expected, the presence of both substituents on ring B in α-configuration favors FXR binding generating potent agonists^25^. Among the two subsets of semisynthetic bile acid derivatives, 3-deoxy-chenodeoxycholane derivatives (subset A) **9** and **10**, differing in the nature of the negative charged side chain end group (sulfate in **9** and carboxylic acid in **10**) transactivate FXR with EC_50_ values of 1.1 μM and 950 nM, respectively. Both values are comparable to that of 6-ECDCA (**6**) (Figure S1) and the above potency was also shared by the corresponding 3β-hydroxyl carboxylic acid **17** (EC_50_ values of 1.3 μM). Of interest are also the results of tauro-conjugation on the potent FXR agonists, carboxylic acids **10** and **17**. As shown in Fig. 6A, tauro-conjugation on the 3-deoxy derivative **10** produces **12**, still a potent FXR agonist whereas tauro-conjugation on the 3β-hydroxyl carboxylic acid **17** is detrimental in term of FXR activation (compound **19** in Fig. 6A). Because endogenous bile acids and semisynthetic bile acid derivatives undergo to extensive liver conjugation at the carboxyl acid end group (tauro-conjugation in mice and glyco-conjugation in human), this result highlights the therapeutical potential of compound **10** in human FXR mediated diseases. Indeed compound **10** is devoid of any activity toward GPBAR1 in agonistic mode (Fig. 8A) but when administered in combination with TLCA, it inhibited TLCA mediated GPBAR1 activation with an IC_50_ value of 11 μM (Fig. 9B). Thus, to the best of our knowledge, compound **10** represents the first example of a 6-ethyl bile acid derivative endowed with FXR agonism/GPBAR1 antagonism.

Further investigating its pharmacological properties, compound **10** at the dose of 15 mg/kg was administered to intact mice. As illustrated in Fig. 10, this agent significantly increased the expression of three canonical FXR target genes in the liver. Indeed, compound **10** increased the expression of OSTα, SHP and BSEP in the liver as early as 6 h after administration. Because these three genes are endowed with canonical FXR-responsive elements in their promoter, their induction is fully consistent with the nature of compound **10** as potent FXR ligand. This view was further confirmed by analysis of bile acids in the blood of mice administered with compound **10**. Indeed, as shown in Fig. 11, exposure to this agent results in a marked reduction of total and single unconjugated bile acids, a feature that is consistent with its FXR agonistic profile. Indeed, activation of FXR in the liver inhibits the activity of CYP7A1, thus causing a reduction of the synthesis of primary bile acids. Further supporting this view, **10** reduced the blood concentrations of 7α-hydroxy-4-cholesten-3-one (Fig. 11D), an intermediate in the synthesis of primary bile acids, that is widely used to indirectly measure CYP7A1 activity *in vivo*. The fact that 7α-hydroxy-4-cholesten-3-one levels were reduced in mice administered with compound **10** further supports this compound as a potent FXR agonist.

In summary, in the present study we describe the synthesis and the *in vitro* and *in vivo* pharmacological characterization of a novel family of compounds including several potent and selective FXR agonists. These novel agents might add utility in the treatment of cholestatic disorders avoiding side effects linked to unwanted activation of GPBAR1.

## Methods

### Chemistry

High-resolution ESI-MS spectra were performed with a Micromass Q-TOF mass spectrometer. NMR spectra were obtained on Varian Inova 400, 500 and 700 NMR spectrometers (^1^H at 400, 500 and 700 MHz, ^13^C at 100, 125 and 175 MHz, respectively) equipped with a SUN microsystem ultra 5 hardware and recorded in CD_3_OD (δ_H_ = 3.31 and δ_C_ = 49.0 ppm) and CDCl_3_ (δ_H_ = 7.26 and δ_C_ = 77.0 ppm). All of the detected signals were in accordance with the proposed structures. Coupling constants (*J* values) are given in Hertz (Hz), and chemical shifts (δ) are reported in ppm and referred to CHD_2_OD and CHCl_3_ as internal standards. Spin multiplicities are given as s (singlet), br s (broad singlet), d (doublet), or m (multiplet). Through-space ^1^H connectivities were evidenced using a NOESY experiment with mixing times of 400 ms, respectively.

HPLC was performed with a Waters Model 510 pump equipped with Waters Rheodine injector and a differential refractometer, model 401. Reaction progress was monitored via thin-layer chromatography (TLC) on Alugram silica gel G/UV254 plates. Silica gel MN Kieselgel 60 (70–230 mesh) from Macherey-Nagel Company was used for column chromatography.

All chemicals were obtained from Sigma-Aldrich, Inc. Solvents and reagents were used as supplied from commercial sources with the following exceptions. Dichloromethane, tetrahydrofuran and triethylamine were distilled from calcium hydride immediately prior to use. Methanol was dried from magnesium methoxide as follow. Magnesium turnings (5 g) and iodine (0.5 g) were refluxed in a small (50–100 mL) quantity of methanol until all of the magnesium has reacted. The mixture was diluted (up to 1 L) with reagent grade methanol, refluxed for 2–3 h then distilled under nitrogen. All reactions were carried out under argon atmosphere using flame-dried glassware.

The purities of compounds were determined to be greater than 95% by HPLC.

### Synthetic procedures

See the Supporting Information.

### Transactivation assay

To evaluate the transcriptional activity of FXR, HepG2 cells were transiently transfected with Fugene HD reagent (Promega) using the followings plasmids: pCMVSPORT-humanFXR, pSG5RXR, p(hsp27)TKLUC and pGL4.70, a plasmid containing the Renilla gene used for luciferase normalization. At 24 h post transfection, cells were stimulated with compounds **7–21** (10 μ M). CDCA (**1**, 10 μ M) and 6-ECDCA (**6**, 1 μ M) were used as a positive controls. Dose-response curves were performed in HepG2 cells transfected as described above and treated with increasing concentrations of **9**, **10**, **15** and **17 (**from 100 nM to 10 μM).

To investigate GPBAR1 activation, HEK-293T cells were transiently transfected with Fugene HD reagent (Promega) using the following vectors: pCMVSPORT6-human GPBAR1, pGL4.29 (Promega), a reporter vector containing a cAMP response element (CRE) cloned upstream to the luciferase reporter gene luc2P and pGL4.70. At 24 h post transfection, cells were stimulated with compounds **7–21** (10 μM) and TLCA (**2**, 10 μM) were used as a positive controls. Dose-response curves were performed in HEK-293T cells transfected as described above and treated with increasing concentrations of **9** and **21 (**from 100 nM to 50 μM). To evaluate the IC_50_ of compound **10**, a dose response curve was performed in HEK-293T cells stimulated with 10 μM TLCA and with increasing concentrations of **10**: range from 5 to 25 μM. At 18 h post stimulations, cellular lysate was assayed for luciferase and renilla activities using the Dual-Luciferase Reporter assay system (E1980, Promega). Luminescence was measured using Glomax 20/20 luminometer (Promega). Luciferase activities were normalized with Renilla activities.

### Direct Interaction on FXR by Alphascreen Technology in a Coactivator Recruitment Assay

Anti-GST-coated acceptor beads were used to capture the GST-fusion FXR-LBD, whereas the biotinylated-SRC-1 peptide was captured by the streptavidin donor beads. Upon illumination at 680 nm, chemical energy is transferred from donor to acceptor beads across the complex streptavidin-donor/Src-1-biotin/GSTFXR-LBD/anti-GST-acceptor and a signal is produced. The assay was performed in white, low-volume, 384-well Optiplates (PerkinElmer) using a final volume of 25 μL containing final concentrations of 10 nM of purified GST-tagged FXR-LBD protein, 30 nM biotinylated Src-1 peptide, 20 μg/mL anti-GST acceptor beads acceptor beads, and 10 μg/mL of streptavidin donor bead (PerkinElmer). The assay buffer contained 50 mM Tris (pH 7.4), 50 mM KCl, 0.1% BSA, and 1 mM DTT. The stimulation times with 1 μL of tested compound (dissolved in 50% DMSO/H_2_O) were fixed to 30 min at room temperature. The concentration of DMSO in each well was maintained at a final concentration of 4%. After the addition of the detection mix (acceptor and donor beads), the plates were incubated in the dark for 4 h at room temperature and then were read in an Envision microplate analyzer (PerkinElmer).

### Animal Studies

All animal experimental procedures were approved by the Ethics Committee of the University of Perugia and by the Italian Health Ministry, according to the Italian guideline for care and use of laboratory animals. C57BL6 mice were treated 6 h with compound **10** (15 mg/kg, ip). At the end of the treatment, mice were sacrificed and liver and blood samples were collected respectively to perform real-time PCR and bile acids quantization.

### RT-PCR

Total RNA was extracted using the TRIzol reagent (Life Technologies). First, 1 μg of RNA was purified of the genomic DNA by DNase I treatment (Life Technologies) and random reverse-transcribed with Superscript II (Life Technologies) in a 20 μL reaction volume. Then 10 ng of cDNA was amplified in a final volume of 20 μL in a mix containing 200 nM of each sense/antisense primers and 10 μL of 2× SYBR Select Master mix (Life Technologies). RT-PCR reactions were performed in duplicate and the thermal cycling conditions were: 2 min at 95 °C, followed by 40 cycles of 95 °C for 20 s, 60 °C for 30 s on Step One Plus Instrument (ABI). The relative mRNA expression was calculated and expressed as 2^−(ΔΔCt)^. Sense and antisense primer sequences were the following: mouse GAPDH, ctgagtatgtcgtggagtctac and gttggtggtgcaggatgcattg; mOSTα, cagtggacatagccctcacc and gaccaaagcagcagaacaca; mBSEP, aaatcggatggtttgactgc and tgacagcgagaatcaccaag; mSHP, tctcttcttccgccctatca and aagggcttgctggacagtta.

### Bile acid and 7α-hydroxy-4-cholesten-3-one determinations

See the Supporting Information.

## Additional Information

**How to cite this article**: Sepe, V. *et al.* Insights on FXR selective modulation. Speculation on bile acid chemical space in the discovery of potent and selective agonists. *Sci. Rep.* **6**, 19008; doi: 10.1038/srep19008 (2016).

## Supplementary Material

Supplementary Information

## Figures and Tables

**Figure 1:**
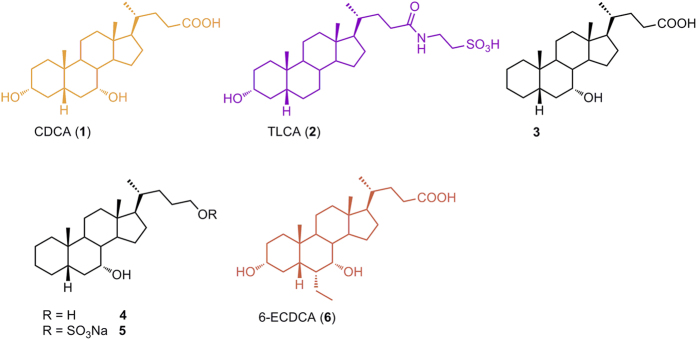
CDCA and TLCA, the most potent endogenous activators of FXR and GPBAR1, respectively. 3-Deoxy-5β-cholane derivatives **3**–**5** as selective FXR ligands and 6-ECDCA, a potent semi-synthetic dual agonist.

**Figure 2:**
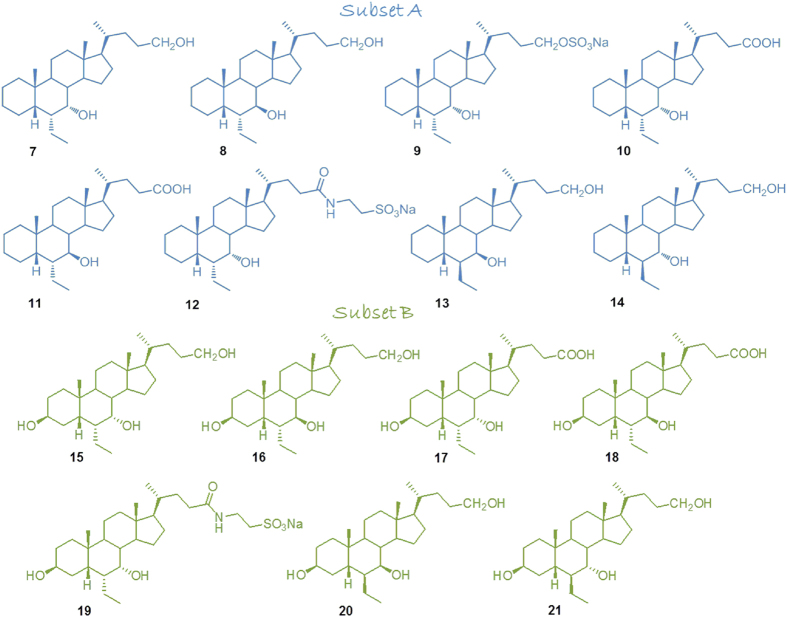
6-Ethylcholane scaffold derivatives generated in this study. Subset A: 3-deoxy-6-ethylchenodeoxycholane derivatives; subset B: 3β-hydroxy-6-ethylchenodeoxycholane derivatives.

**Figure 5:**
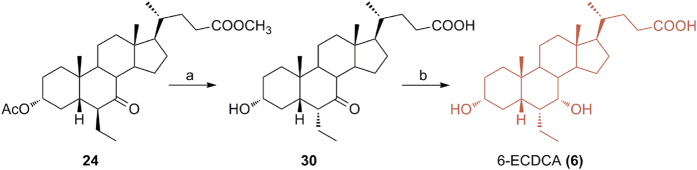
Preparation of the reference compound, 6-ECDCA (6). *Reagents and conditions*: (**a**) NaOH, MeOH:H_2_O 1:1 v/v; (**b**) LiBH_4_, MeOH dry, THF, 0 °C, 69% over two steps.
