# Supplementary material for: Insights on FXR selective modulation. Speculation on bile acid chemical space in the discovery of potent and selective agonists
Source: Sci Rep. 2016 Jan 7;6:19008. doi: 10.1038/srep19008 (PMC4704022; doi:10.1038/srep19008)
Supplement: Supplementary Information [file srep19008-s1.pdf]

Supporting Information

Insights on FXR selective modulation. Speculation on bile acid chemical space in the discovery of potent and selective agonists

Valentina Sepe,[1] Carmen Festa,[1] Barbara Renga,[2] Adriana Carino,[2] Sabrina Cipriani,[2] Claudia Finamore,[1] Dario Masullo,[1] Federica del Gaudio,[3] Maria Chiara Monti,[3] Stefano Fiorucci,[2] and Angela Zampella[1]*

[1]Department of Pharmacy, University of Naples "Federico II", Via D. Montesano, 49, I-80131 Naples, Italy
[2]Department of Surgery and Biomedical Sciences, Nuova Facoltà di Medicina, P.zza L. Severi 1-06132 Perugia, Italy
[3]Department of Pharmacy, University of Salerno, Via Giovanni Paolo II, 132, 84084, Fisciano (Salerno), Italy

*A.Z.: phone, +39 081678525; fax, +39 081678552; e-mail, angela.zampella@unina.it

Table of contents:

- Synthetic procedures S2
- Bile acids determination S12
- Determination of 7α-hydroxy-4-cholesten-3-one S14
- ^{1}H-NMR spectrum of compound **7** S15
- ^{1}H-NMR spectrum of compound **8** S16
- ^{1}H-NMR spectrum of compound **9** S17
- ^{1}H-NMR spectrum of compound **10** S18
- ^{1}H-NMR spectrum of compound **11** S19
- ^{1}H-NMR spectrum of compound **12** S20
- ^{1}H-NMR spectrum of compound **13** S21
- ^{1}H-NMR spectrum of compound **14** S22
- ^{1}H-NMR spectrum of compound **15** S23
- ^{1}H-NMR spectrum of compound **16** S24
- ^{1}H-NMR spectrum of compound **17** S25
- ^{1}H-NMR spectrum of compound **18** S26
- ^{1}H-NMR spectrum of compound **19** S27
- ^{1}H-NMR spectrum of compound **20** S28
- ^{1}H-NMR spectrum of compound **21** S29
- Figure S1. Concentration-response curve of 6-EDCA (**6**) on FXR S30

Synthetic procedures.

Methyl 3α-acetoxy-7-keto-5β-cholan-24-oate (22) To a solution of 7-ketolithocholic acid (5 g, 12.8 mmol), dissolved in 100 mL of dry methanol was added *p*-toluenesulfonic acid (11 g, 64.1 mmol). The solution was left to stand at room temperature for 2 h. The mixture was quenched by addition of $NaHCO_3$ saturated solution. After the evaporation of the methanol, the residue was extracted with EtOAc (3x150 mL). The combined extract was washed with brine, dried with Na_2SO_4, and evaporated to give the methyl esters amorphous solid (5.13 g, quantitative yield).

At the solution of the methyl ester (5.13 g, 12.7 mmol) in dry pyridine (100 mL), an excess of acetic anhydride was added. When the reaction was complete, the pyridine was concentrated under vacuum. The residue was poured into cold water (100 mL) and extracted with ethyl acetate (3 × 150 mL). The combined organic phases were dried (Na_2SO_4) and concentrated to give a residue that was further purified by flash chromatography on silica gel using hexane/ethyl acetate 8:2 and 0.5% of triethylamine as eluent (4.8 g of **22** as a white solid, 84% yield over two steps).

Methyl 3α-acetoxy-6-ethylidene-7-keto-5β-cholan-24-oate (23). To a solution of diisopropylamine (23 mL, 0.16 mol) in dry THF (50 mL) was added dropwise a solution of *n*-butyllithium (60 mL, 2.5 M in hexane, 0.15 mol) at -78 °C. After 30 min, trimethylchlorosilane (27.1 mL, 0.21 mol) was added. After additional 30 min, a solution of compound **22** (4.8 g, 10.7 mmol) in dry THF (70 mL) was added. The reaction was stirred at -78 °C for an additional 45 min and then triethylamine (54 mL, 0.38 mol) was added. After 1 h, the reaction mixture was allowed to warm to -20 °C, treated with aqueous saturated solution of $NaHCO_3$ (100 mL) and brought up to room temperature in 2 h. The aqueous phase was extracted with ethyl acetate (3 × 50 mL). The combined organic phases were washed then with saturated solution of $NaHCO_3$, water and brine. After drying over anhydrous Na_2SO_4, the residue was evaporated under vacuum to give 6 g of yellow residue, that was diluted in dry CH_2Cl_2 (50 mL) and cooled at -78 °C. At this stirred solution acetaldehyde (3 mL, 53 mmol) and $BF_3{\cdot}OEt_2$ (13.5 mL, 0.107 mol) were added dropwise. The reaction mixture was stirred for 2 h at -60 °C and allowed to warm to room temperature. The mixture was quenched with saturated aqueous

solution of $NaHCO_3$ and extracted with CH_2Cl_2. The combined organic phases were washed with brine, dried over anhydrous Na_2SO_4 and concentrated under *vacuum*.

Purification by silica gel (hexane-ethyl acetate 9:1 and 0.5% TEA) gave compound **23** (4.1 g, 80% over two steps). NMR analysis demonstrated a diasteromeric ratio E/Z >95%. The *E* configuration at the exocyclic double bond was established by dipolar coupling H_3-26 (δ 1.67)/H-5 (δ 2.62) in Noesy spectrum (400 MHz, mixing time 400 ms).

Selected ^{1}H NMR (400 MHz, $CDCl_3$): δ 6.16 (1H, q, *J* = 7.0 Hz, H-25), 4.74 (1H, m, H-3), 3.64 (3H, s, $COOCH_3$), 2.62 (1H, dd, *J* = 13.0, 3.6 Hz, H-5), 1.98 (3H, s, $COCH_3$), 1.67 (3H, d, *J* = 7.0 Hz, H_3-26), 1.00 (3H, s, H_3-19), 0.92 (3H, d, *J* = 6.0 Hz, H_3-21), 0.67 (3H, s, H_3-18); ^{13}C NMR (100 MHz, $CDCl_3$): δ 204.5, 174.6, 170.7, 143.1, 130.2, 72.5, 54.5, 51.4, 50.7, 48.6, 45.2, 43.5, 39.1, 38.9, 35.1, 34.9, 34.1, 33.4, 31.0, 30.9, 28.4, 25.9 (2C), 22.8, 21.4, 21.2, 18.4, 12.7, 12.2. HR ESIMS *m/z* 473.3271 $[M+H]^+$, $C_{29}H_{45}O_5$ requires 473.3267.

Methyl 3α-acetoxy-6β-ethyl-7-keto-5β-cholan-24-oate (24). A solution of **23** (4.0 g, 8.5 mmol) in THF dry/MeOH dry (100 mL, 1:1 v/v) was hydrogenated in presence of $Pd(OH)_2$ 20% wt on activated carbon (100 mg) degussa type. The mixture was transferred to a standard PARR apparatus and flushed with nitrogen and then with hydrogen several times. The apparatus was shacked under 50 psi of H_2. The reaction was stirred at room temperature for 8 h. The catalyst was filtered through Celite, and the recovered filtrate was concentrated under vacuum to give **24** (4.0 g, quantitative yield). The β configuration of ethyl group at C-6 was determined by dipolar couplings H_3-26 (δ 0.83)/ H_3-19 (δ 1.22) and H-8 (δ 2.56)/H-25 (δ 1.83) in Noesy spectrum (400 MHz, mixing time 400 ms).

Selected ^{1}H NMR (400 MHz, CD_3OD): δ 4.65 (1H, m, H-3), 3.66 (3H, s, $COOCH_3$), 2.56 (1H, t, *J* = 11.5 Hz, H-8), 2.35 (1H, m, H-23a), 2.22 (1H, m, H-23b), 1.99 (3H, s, $COCH_3$), 1.22 (3H, s, H_3-19), 0.92 (3H, d, *J* = 6.3 Hz, H_3-21), 0.83 (3H, t, *J* = 7.2 Hz, H_3-26), 0.67 (3H, s, H_3-18). ^{13}C NMR (100 MHz,CD_3OD): δ 214.7, 174.3, 170.2, 72.6, 61.7, 54.8, 51.3, 49.0, 48.5, 45.3, 42.7, 42.3 (2C), 38.6,

35.4, 35.1, 35.0, 31.0, 30.8, 28.0 (2C), 26.4, 25.7, 24.7, 21.3, 21.1, 18.2, 12.9, 11.9. HR ESIMS *m/z* 475.3430 $[M+H]^+$, $C_{29}H_{47}O_5$ requires 475.3423.

Methyl 6α-ethyl-7-keto-5β-cholan-24-oate (26). Compound **24** (500 mg, 1.05 mmol) was treated with MeONa in methanol to obtain deacetylation at C-3 and inversion at C-6. Tosylation, in the same operative condition previously described,[1] furnished 620 mg of **25** (quantitative yield over two steps) that was subjected to the next step without further purification.

Lithium bromide (148 mg, 1.7 mmol) and lithium carbonate (125 mg, 1.7 mmol) were added to a solution of 6α-ethyl-3α-tosyloxy-7-keto-5β-cholan-24-oate (500 mg, 0.85 mmol) in dry DMF (30 mL), and the mixture was refluxed for 2 h. After cooling to room temperature, the mixture was slowly poured into 10% HCl solution (20 mL) and extracted with CH_2Cl_2 (3 × 50 mL). The combined organic layer was washed successively with water, saturated $NaHCO_3$ solution and water, and then dried over anhydrous $MgSO_4$ and evaporated to dryness to give 400 mg of oleos residue (quantitative yield), that was subjected to next step without any purification.

Hydrogenation on $Pd(OH)_2$ in the same operative condition previously described furnished 312 mg of **26** (88% over two steps).

6α-ethyl-7α-hydroxy-5β-cholan-24-ol (7) and 6α-ethyl-7β-hydroxy-5β-cholan-24-ol (8). Dry methanol (70 μL, 1.7 mmol) and $LiBH_4$ (850 μL, 2 M in THF, 1.7 mmol) were added to a solution of the compound **26** (100 mg, 0.24 mmol) in dry THF (5 mL) at 0 °C under argon and the resulting mixture was stirred for 5 h at 0 °C. The mixture was quenched by addition of NaOH (1 M, 480 μL) and then allowed to warm to room temperature. Ethyl acetate was added and the separated aqueous phase was extracted with ethyl acetate (3 ×15 mL). The combined organic phases were washed with water, dried (Na_2SO_4) and concentrated to give a mixture of alcohols **7** and **8**. HPLC purification on a Nucleodur 100-5 C18 (5 μm; 10 mm i.d. x 250 mm) with $MeOH/H_2O$ (92:8) as eluent (flow rate 3 mL/min), gave 64 mg of 6α-ethyl-7α-hydroxy-5β-cholan-24-ol **7** (69%, t_R= 31 min) and a small amount of 6α-ethyl-7β-hydroxy-5β-cholan-24-ol **8** (8 mg, t_R= 24.8 min).

6α-ethyl-7α-hydroxy-5β-cholan-24-ol (7). Selected ^{1}H NMR (400 MHz CD_3OD): δ 3.65 (1H, br s, H-7), 3.51 (2H, m, H_2-24), 0.97 (3H, d, *J* = 6.3 Hz, H_3-21), 0.92 (3H, s, H_3-19), 0.89 (3H, t, *J* = 7.3 Hz, H_3-26), 0.71 (3H, s, H_3-18). ^{13}C NMR (100 MHz CD_3OD): δ 71.6, 63.6, 57.6, 51.8, 48.7, 43.7, 43.3, 41.5, 41.1, 39.3, 37.5, 37.0, 34.6, 33.2, 30.3, 29.4, 28.8, 25.1, 24.6 (2C), 23.5, 22.5, 22.0, 19.2, 12.3, 12.1. HR ESIMS *m/z* 391.3579 $[M+H]^+$, $C_{26}H_{47}O_2$ requires 391.3576.

6α-ethyl-7β-hydroxy-5β-cholan-24-ol (8). Selected ^{1}H NMR (500 MHz, CD_3OD): δ 3.51 (2H, m, H_2-24), 3.07 (1H, t, *J* = 10.0 Hz, H-7), 0.96 (3H, d, *J* = 6.6 Hz, H_3-21), 0.96 (3H, t, *J* = 7.3 Hz, H_3-26), 0.95 (3H, s, H_3-19), 0.71 (3H, s, H_3-18). Selected ^{13}C NMR (100 MHz CD_3OD): δ 76.3, 63.6, 57.9, 56.8, 46.3, 45.3, 45.0, 44.7, 41.7, 41.1, 38.8, 37.0, 36.3, 33.3, 30.3, 28.1, 27.9 (2C), 25.0 (2C), 22.0, 21.9 (2C), 19.4, 12.7, 11.6. HR ESIMS *m/z* 391.3581 $[M+H]^+$, $C_{26}H_{47}O_2$ requires 391.3576.

6α-ethyl-7α-hydroxy-5β-cholan-24-yl 24-sodium sulfate (9). Sulfation on C-24 on a small aliquot of diol **7** (20 mg, 0.05 mmol) was performed in the same operative conditions previously described.[1,2] RP18/HPLC on a Nucleodur 100-5 C18 (5 μm; 10 mm i.d. x 250 mm) with MeOH/H_2O (82:18) as eluent (flow rate 3 mL/min) afforded compound **9** (t_R= 14.2 min) as sodium salt. Selected ^{1}H NMR (400 MHz CD_3OD): δ 3.96 (2H, t, *J* = 6.3 Hz, H_2-24), 3.64 (1H, br s, H-7), 0.96 (3H, d, *J* = 6.6 Hz, H_3-21), 0.91 (3H, s, H_3-19), 0.88 (3H, t, *J* = 7.4 Hz, H_3-26), 0.69 (3H, s, H_3-18). HR ESIMS *m/z* 469.2991 $[M-H]^-$, $C_{26}H_{45}O_5S$ requires 469.2988.

6α-ethyl-7α-hydroxy-5β-cholan-24-oic acid (10) and 6α-ethyl-7β-hydroxy-5β-cholan-24-oic acid (11). Compound **26** (200 mg, 0.48 mmol) was hydrolyzed with NaOH (96 mg, 2.4 mmol) in a solution of MeOH:H_2O 1:1 v/v (10 mL). The mixture was stirred for 4 h at reflux. The resulting solution was then acidified with HCl 6N and extracted with ethyl acetate (3 × 50 mL). The collected organic phases were washed with brine, dried over Na_2SO_4 anhydrous and evaporated under reduced pressure to give the carboxylic acid intermediate**.**

Crude carboxylic acid intermediate (190 mg, 0.47 mmol) was treated with $LiBH_4$ (1.65 mL, 2M in THF, 3.3 mmol) and MeOH (133 μL, 3.3 mmol) in THF dry (5 mL). Purification by silica gel

(CH_2Cl_2-MeOH 99:1) furnished 157 mg of 6α-ethyl-7α-hydroxy-5β-cholan-24-oic acid (**10**, 83%). In same embodiments $LiBH_4$ treatment after alkaline hydrolysis produced small amounts (about 10%) of 6α-ethyl-7β-hydroxy-5β-cholan-24-oic acid (**11**) that was isolated by HPLC purification on a Nucleodur 100-5 C18 (5 μm; 10 mm i.d. x 250 mm) with MeOH/H_2O (88:12) as eluent (flow rate 3 mL/min, t_R= 16 min).

6α-ethyl-7α-hydroxy-5β-cholan-24-oic acid (10). Selected ^{1}H NMR (400 MHz CD_3OD): δ 3.65 (1H, brs, H-7), 2.34 (1H, m, H-23a), 2.20 (1H, m, H-23b), 0.96 (3H, d, *J* = 6.3 Hz, H_3-21), 0.92 (3H, s, H_3-19), 0.89 (3H, t, *J* = 7.4 Hz, H_3-26), 0.70 (3H, s, H_3-18). ^{13}C NMR (100 MHz CD_3OD): δ 178.0, 71.6, 57.4, 51.7, 48.7, 43.8, 43.3, 41.5, 41.1, 39.3, 37.4, 36.8, 34.6, 32.5 (2C), 29.3, 28.8, 25.1, 24.6 (2C), 23.5, 22.5, 22.0, 18.8, 12.2, 12.1. HR ESIMS *m/z* 403.3214 $[M-H]^-$, $C_{26}H_{43}O_3$ requires 403.3212.

6α-ethyl-7β-hydroxy-5β-cholan-24-oic acid (11). Selected ^{1}H NMR (500 MHz CD_3OD): δ 3.08 (1H, t, *J* = 9.6 Hz, H-7), 2.32 (1H, m, H-23a), 2.20 (1H, m, H-23b), 0.96 (3H, d, *J* = 6.2 Hz, H_3-21), 0.95 (3H, s, H_3-19), 0.85 (3H, t, *J* = 7.0 Hz, H_3-26), 0.70 (3H, s, H_3-18). HR ESIMS *m/z* 403.3217 $[M-H]^-$, $C_{26}H_{43}O_3$ requires 403.3212.

6α-ethyl-7α-hydroxy-5β-cholan-24-oyl taurine sodium sulfate (12). An aliquot of **10** (10 mg, 0.024 mmol) in DMF dry (5 mL) was treated with DMT-MM (20.5 mg, 0.07 mmol) and triethylamine (83μL, 0.6 mmol) and the mixture was stirred at room temperature for 10 min. Then to the mixture was added taurine (18 mg, 0.14 mmol). After 3 h, the reaction mixture was concentrated under vacuo and dissolved in water (5 mL). Purification on C18 silica gel column and then HPLC on a Nucleodur 100-5 C18 (5 μm; 10 mm i.d. x 250 mm) with MeOH/H_2O (83:17) as eluent (flow rate 3 mL/min), gave 4.5 mg of 6α-ethyl-7α-hydroxy-5β-cholan-24-oyl taurine sodium sulfate (**12**) (t_R= 10 min). Selected ^{1}H NMR (400 MHz CD_3OD): δ 3.65 (1H, br s, H-7), 3.58 (2H, t, *J* = 7.0 Hz, CH_2-N), 2.96 (2H, t, *J* = 9.6 Hz, CH_2-S), 2.25 (1H, m, H-23a), 2.10 (1H, m, H-23b), 0.97 (3H, d, *J* = 6.4 Hz, H_3-

21), 0.92 (3H, s, H_3-19), 0.89 (3H, t, *J* = 7.1 Hz, H_3-26), 0.70 (3H, s, H_3-18). HR ESIMS *m/z* 510.3257 $[M-H]^-$, $C_{28}H_{48}NO_5S$ requires 510.3253.

Methyl 6β-ethyl-7-keto-5β-cholan-24-oate (28). Compound **28** (400 mg, 94% over two steps) was synthesized, starting from compound **27** (600 mg, 1.02 mmol), by an analogous procedure to that detailed above for compound **26**. Compound **27** (620 mg, quantitative yield over two steps) was obtained from **24** (500 mg, 1.05 mmol) by an analogous procedure to that detailed above for compound **25**, except for reaction time (2 h) of the deacetylation step.

Selected ^{1}H NMR (400 MHz, CD_3OD): δ 3.63 (3H, s, $COOCH_3$), 2.53 (1H, t, *J* = 11.4 Hz, H-8), 2.33 (1H, m, H-23a), 2.19 (1H, m, H-23b), 1.18 (3H, s, H_3-19), 0.89 (3H, d, *J* = 6.2 Hz, H_3-21), 0.81 (3H, t, *J* = 7.4 Hz, H_3-26), 0.64 (3H, s, H_3-18); ^{13}C NMR (100 MHz, CD_3OD): δ 217.0, 174.9, 62.4, 54.7, 51.5, 50.7, 48.7, 45.7, 43.2, 42.5, 38.8, 37.5, 36.3, 35.2, 31.0, 30.9, 30.3, 28.2, 26.7 (2C), 26.3, 24.9, 21.4, 20.4, 18.3, 13.0, 12.0; HR ESIMS *m/z* 417.3373 $[M+H]^+$, $C_{27}H_{43}O_3$ requires 417.3369.

6β-ethyl-7β-hydroxy-5β-cholan-24-ol (13) and 6β-ethyl-7α-hydroxy-5β-cholan-24-ol (14). To a methanol solution of **28** (350 mg, 0.84 mmol), a large excess of $NaBH_4$ was added at 0 °C. The mixture was left at room temperature for 2 h and then water and MeOH were added dropwise during a period of 15 min at 0 °C with effervescence being observed. After evaporation of the solvents, the residue was diluted with water and extracted with ethyl acetate (3x50 mL). The combined extract was washed with brine, dried with Na_2SO_4, and evaporated to give 1.3 g of a crude residue that was subjected to the next step without further purification. The crude residue was treated with $LiBH_4$ (2M in THF) in the same operative condition described for the synthesis of compounds **7** and **8**. HPLC purification on a Nucleodur 100-5 C18 (5 μm; 10 mm i.d. x 250 mm) with MeOH/H_2O (92:8) as eluent (flow rate 3 mL/min), furnished 180 mg of 6β-ethyl-7β-hydroxy-5β-cholan-24-ol (**13**, 54%, t_R= 25 min) and 75.4 mg of 6β-ethyl-7α-hydroxy-5β-cholan-24-ol (**14**, 23%, t_R= 13 min).

6β-ethyl-7β-hydroxy-5β-cholan-24-ol (13). Selected ^{1}H NMR (700 MHz CD_3OD): δ 3.67 (1H, dd, *J* = 8.7, 4.7 Hz, H-7), 3.51 (2H, m, H_2-24), 0.98 (3H, s, H_3-19), 0.97 (3H, d, *J* = 6.6 Hz, H_3-21), 0.96

(3H, t, J = 7.4 Hz, H_3-26), 0.71 (3H, s, H_3-18). ^{13}C NMR (175 MHz CD_3OD): δ 75.5, 63.8, 57.6, 56.5, 44.2, 43.7, 42.8, 41.0, 40.9, 40.8, 38.2 (2C), 36.9, 34.4, 32.8, 29.7, 28.9, 27.0, 26.1, 24.7, 22.2, 22.0 (2C), 19.2, 13.9, 12.3. HR ESIMS *m/z* 391.3580 $[M+H]^+$, $C_{26}H_{47}O_2$ requires 391.3576.

6β-ethyl-7α-hydroxy-5β-cholan-24-ol (14). Selected ^1H NMR (700 MHz CD_3OD): δ 3.59 (1H, s, H-7), 3.51 (2H, m, H_2-24), 2.23 (1H, dq, J = 13.9, 4.0 Hz, H-4a), 0.97 (3H, d, J = 6.6, H_3-21), 0.95 (3H, t, J = 7.1 Hz, H_3-26), 0.94 (3H, s, H_3-19), 0.70 (3H, s, H_3-18). ^{13}C NMR (175 MHz CD_3OD): δ 73.1, 63.2, 57.3, 52.8, 51.4, 49.4, 43.8, 41.3, 39.7, 37.4 (2C), 37.2, 34.2, 32.6 (2C), 29.9, 29.5, 28.9, 28.3, 27.3, 24.5, 21.7, 21.2, 18.8, 14.3, 12.3. HR ESIMS *m/z* 391.3578 $[M+H]^+$, $C_{26}H_{47}O_2$ requires 391.3576.

Methyl 6α-ethyl-3β-hydroxy-7-keto-5β-cholan-24-oate (29). A solution of **27** (900 mg, 1.5 mmol) and CH_3COOK (147 mg, 1.53 mmol) dissolved in water (2 mL) and N,N'-dimethylformamide (DMF, 10 mL) was refluxed for 2 h. The solution was cooled at room temperature and then ethyl acetate and water were added. The separated aqueous phase was extracted with ethyl acetate (3 ×30 mL). The combined organic phases were washed with water, dried (Na_2SO_4) and evaporated to dryness to give 1.0 g of mixture. Purification by silica gel (hexane-ethyl acetate 7:3 and 0.5% TEA) gave 500 mg of intermediate as oily oil. C-6 inversion in the same operative condition as described for the synthesis of compound **25**, furnished compound **29** (500 mg, 74% over two steps). Selected ^1H NMR (400 MHz CD_3OD): δ 3.96 (1H, m, H-3), 3.64 (3H, s, $COOCH_3$), 2.85 (1H, dd, J = 5.6, 12.3 Hz, H-6), 2.50 (1H, t, J = 11.2 Hz, H-8), 2.33 (1H, m, H-23a), 2.20 (1H, m, H-23b), 1.27 (3H, s, H_3-19), 0.95 (3H, d, J = 6.4 Hz, H_3-21), 0.81 (3H, t, J = 7.2 Hz, H_3-26), 0.71 (3H, s, H_3-18); ^{13}C NMR (100 MHz CD_3OD): δ 213.4, 174.8, 65.9, 54.8, 51.8, 51.2, 50.0, 49.0, 45.7, 43.2, 42.7, 39.0, 36.2, 35.2, 31.0 (2C), 29.2, 29.0, 28.3, 27.2, 24.6, 24.0, 22.1, 18.7, 18.4, 12.0 (2C). HR ESIMS *m/z* 433.3322 $[M + H]^+$, $C_{27}H_{45}O_4$ requires 433.3318.

6α-ethyl-3β,7α-dihydroxy-5β-cholan-24-ol (15) and 6α-ethyl-3β,7β-dihydroxy-5β-cholan-24-ol (16). Intermediate **29** (500 mg, 1.16 mmol) was treated with $LiBH_4$ as previously described. HPLC

purification on a Nucleodur 100-5 C18 (5 μm; 10 mm i.d. x 250 mm) with $MeOH/H_2O$ (88:12) as eluent (flow rate 3 mL/min), gave 250 mg of compound **15** as a white solid (53%, t_R= 12.6 min) and a small amount of compound **16** (23 mg, 5%, tr=8.2 min).

6α-ethyl-3β,7α-dihydroxy-5β-cholan-24-ol (15). Selected ^1H NMR (500 MHz CD_3OD): δ 3.97 (1H, brs, H-3), 3.66 (1H, brs, H-7), 3.51 (2H, m, H_2-24), 0.96 (3H, d, *J* = 6.6 Hz, H_3-21), 0.94 (3H, s, H_3-19), 0.91 (3H, t, *J* = 7.5 Hz, H_3-26), 0.70 (3H, s, H_3-18). ^{13}C NMR (125 MHz, CD_3OD): δ 71.4, 67.4, 63.6, 57.6, 51.7, 43.7, 42.8, 41.5, 41.2, 41.1, 37.1 (2C), 33.8, 33.2, 31.3 (2C), 30.3, 29.4, 28.3, 24.6, 24.2, 23.3, 22.3, 19.2, 12.7, 12.1. HR ESIMS *m/z* 407.3531 $[M+H]^+$, $C_{26}H_{47}O_3$ requires 407.3525.

6α-ethyl-3β,7β-dihydroxy-5β-cholan-24-ol (16). Selected ^1H NMR (500 MHz CD_3OD): δ 4.01 (1H, brs, H-3), 3.51 (2H, m, H_2-24), 3.05 (1H, t, *J* = 9.7 Hz, H-7), 0.97 (3H, s, H_3-19), 0.96 (3H, d, *J* = 6.4 Hz, H_3-21), 0.88 (3H, t, *J* = 7.6 Hz, H_3-26), 0.72 (3H, s, H_3-18). ^{13}C NMR (100 MHz CD_3OD): δ 76.3, 67.1, 63.6, 57.8, 56.8, 45.0, 44.8, 44.5, 41.7, 40.3, 39.1, 37.0, 35.9, 33.0, 31.2, 30.2, 29.8, 28.4, 28.0, 27.9, 24.8, 22.9, 21.8, 19.3, 12.8, 11.6. HR ESIMS *m/z* 407.3529 $[M+H]^+$, $C_{26}H_{47}O_3$ requires 407.3525.

6α-ethyl-3β,7α-dihydroxy-5β-cholan-24-oic acid (17) and 6α-ethyl-3β,7β-dihydroxy-5β-cholan-24-oic acid (18). Compounds **17** and **18** (215 mg, 74% over two steps) was synthesized, starting from compound **29** (320 mg, 0.74 mmol), by an analogous procedure to that detailed above for compounds **10** and **11**.

HPLC purification on a Nucleodur100-5 C18 (5 μm; 10 mm i.d. x 250 mm) with $MeOH/H_2O$ (88:12) as eluent (flow rate 3 mL/min), gave 208 mg of **17** as a white solid (65%, t_R= 11 min). In same embodiments $LiBH_4$ treatment after alkaline hydrolysis produced small amounts (about 10%) of **18** that was isolated by HPLC purification on a Nucleodur 100-5 C18 (5 μm; 10 mm i.d. x 250 mm) with $MeOH/H_2O$ (88:12) as eluent (flow rate 3 mL/min, 28 mg of **18**, 9%, t_R= 8 min).

6α-ethyl-3β,7α-dihydroxy-5β-cholan-24-oic acid (17). Selected ^{1}H NMR (400 MHz CD_3OD): δ 3.97 (1H, brs, H-3), 3.67 (1H, br s, H-7), 2.33 (1H, m, H-23a), 2.21 (1H, m, H-23b), 0.96 (3H, d, *J* = 6.5 Hz, H_3-21), 0.94 (3H, s, H_3-19), 0.91 (3H, t, *J* = 7.6 Hz, H_3-26), 0.70 (3H, s, H_3-18). ^{13}C NMR (100 MHz CD_3OD): δ 178.3, 71.3, 67.5, 57.4, 51.7, 43.8, 42.8, 41.5, 41.2, 41.0, 37.0, 36.7, 33.8, 32.4, 32.0, 31.1 (2C), 29.3, 28.3, 24.6, 24.2, 23.3, 22.2, 18.8, 12.3, 12.2. HR ESIMS *m/z* 419.3169 $[M-H]^-$, $C_{26}H_{43}O_4$ requires 419.3167.

6α-ethyl-3β,7β-dihydroxy-5β-cholan-24-oic acid (18). Selected ^{1}H NMR (500 MHz CD_3OD): δ 4.01 (1H, brs, H-3), 3.06 (1H, t, *J* = 9.7 Hz, H-7), 2.32 (1H, m, H-23a), 2.19 (1H, m, H-23b), 0.97 (3H, s, H_3-19), 0.96 (3H, d, ovl, H_3-21), 0.87 (3H, t, *J* = 7.7 Hz, H_3-26), 0.71 (3H, s, H_3-18). HR ESIMS *m/z* 419.3169 $[M-H]^-$, $C_{26}H_{43}O_4$ requires 419.3167.

6α-ethyl-3β,7α-dihydroxy-5β-cholan-24-oyl taurine sodium sulfate (19). Compound **19** was synthesized, starting from compound **17** (10 mg, 0.023 mmol), by an analogous procedure to that detailed above for compound **12**. Selected ^{1}H NMR (500 MHz CD_3OD): δ 3.97 (1H, brs, H-3), 3.67 (1H, br s, H-7), 3.59 (2H, t, *J* = 6.8 Hz, CH_2-N), 2,96 (2H, t, *J* = 6.8 Hz, CH_2-S), 0.97 (3H, d, *J* = 6.4 Hz, H_3-21), 0.95 (3H, s, H_3-19), 0.91 (3H, t, *J* = 7.1 Hz, H_3-26), 0.70 (3H, s, H_3-18). HR ESIMS *m/z* 526.3206 $[M-H]^-$, $C_{28}H_{48}NO_6S$ requires 526.3202.

6β-ethyl-3β,7β-dihydroxy-5β-cholan-24-ol (20) and 6β-ethyl-3β,7α-dihydroxy-5β-cholan-24-ol (21). Compound **27** was treated with CH_3COOK as previously described for compound **29**. $NaBH_4/LiBH_4$ reduction on the corresponding 7-keto intermediate (100 mg, 0.23 mmol) in the same operative conditions described for the synthesis of compounds **13** and **14** afforded a mixture whose HPLC purification on a Nucleodur 100-5 C18 (5 μm; 10 mm i.d. x 250 mm) with $MeOH/H_2O$ (88:12) as eluent (flow rate 3 mL/min), gave 48.3 mg of **20** (52 % over two steps, t_R= 11 min) and 20.7 mg of **21** (22 % over two steps, t_R= 13 min).

6β-ethyl-3β,7β-dihydroxy-5β-cholan-24-ol (20). Selected ^{1}H NMR (700 MHz CD_3OD): δ 3.59 (1H, brs, H-3), 3.57 (1H, dd,*J* = 12.6, 2.3Hz, H-7), 3.51 (2H, m, H_2-24), 0.98 (3H, s, H_3-19), 0.96 (3H,

ovl, H_3-21), 0.96 (3H, t, ovl, H_3-26), 0.70 (3H, s, H_3-18). ^{13}C NMR (175 MHz CD_3OD): δ 75.2, 68.3, 63.6, 58.3, 57.1, 45.7 (2C), 44.2, 41.8 (2C), 41.2, 40.0, 37.0, 35.9, 33.3, 31.1, 30.3, 29.4 (2C), 26.6 (2C), 23.2 (2C), 19.3, 13.0, 12.3. HR ESIMS *m/z* 407.3530 $[M+H]^+$, $C_{26}H_{47}O_3$ requires 407.3525.

6β-ethyl-3β,7α-dihydroxy-5β-cholan-24-ol (21). Selected ^{1}H NMR (700 MHz CD_3OD): δ 3.91 (1H, brs, H-3), 3.60 (1H, br s, H-7), 3.51 (2H, m, H_2-24), 2.45 (1H, t, J = 13.3 Hz, H-4a), 0.97 (3H, s, H_3-19), 0.97 (3H, ovl, H_3-21), 0.95 (3H, t, J = 7.4 Hz, H_3-26), 0.71 (3H, s, H_3-18). ^{13}C NMR (175 MHz CD_3OD): δ 72.8, 67.4, 63.4, 57.2, 51.3, 51.2, 43.2, 41.6, 40.5, 37.3, 37.1 (2C), 36.9, 34.0, 33.3, 32.1, 30.3, 29.3, 28.9, 28.6, 26.3, 24.9, 22.0, 19.3, 13.8, 12.1. HR ESIMS *m/z* 407.3528 $[M+H]^+$, $C_{26}H_{47}O_3$ requires 407.3525.

6α-ethyl-chenodeoxycholic acid (6). Compound **24** (500 mg, 1.05 mmol) was hydrolyzed with NaOH (208 mg, 5.2 mmol) in a solution of MeOH:H_2O 1:1 v/v (10 mL), as previously described. The mixture was stirred for 5 h at reflux. The resulting solution was then acidified with HCl 6N and extracted with ethyl acetate (3 x 50 mL). The collected organic phases were washed with brine, dried over Na_2SO_4 anhydrous and evaporated under reduced pressure to give the carboxylic acid intermediate **30.** This compound was subjected to the $LiBH_4$ reduction of the C7-carbonyl group. Purification gave 305 mg of 6-ECDCA **(6)** as a white solid (69% over two steps).

Bile acids determination

Sample preparation. The stock solutions of the individual tauro-conjugated and un-conjugated bile acids were prepared separately in methanol at a concentration of 1 mg/mL. All stock solutions were stored at −20 °C. Calibration standards were prepared by combining appropriate volumes of each bile acid stock solution and methanol. The calibration range was from 10 nM to 100 μM of each bile acid in the final solution. Mice serum sample aliquots of 100 μL were deproteinized with 1 mL of cold acetonitrile with 5% of NH_4OH vortexing for 1 min. After centrifugation at 16000 g for 10 min, the clear supernatant was transferred to a new vial, snap frozen and lyophilized. The sample was then re-dissolved in methanol–water (2:1, v/v) for tauro-conjugated bile acids determination and in methanol-ammonium acetate 10 mM with 0.005% formic acid (3:2, v/v) for un-conjugated bile acids determination. A bile acids extraction yield of 95% has been estimated.

Liquid chromatography and mass spectrometry. For LC–MS/MS analysis, chromatographic separation was carried out on the HPLC–MS system LTQ XL ThermoScientific equipped with Accelera 600 Pump and Accelera AutoSampler system. The mixture was separated on a Jupiter 5μC18 column from Phenomenex (150 x 2.00 mm). Tauro-conjugated bile acids were separated at a flow rate of 200 μl/min using a methanol–aqueous ammonium acetate (NH_4OAc) gradient. Mobile phase A was 5% methanol in water containing 2 mM ammonium acetate at pH 7, mobile phase B was methanol, containing ammonium acetate at 2 mM. The gradient started at 30 % B and increased to 100% B in 20 min, kept at 100% B for 5 min then decreased to 30% B in 1 min and kept at 30% B for 10 min. ESI was performed in negative ion mode, the ion source temperature was set at 280 °C. The tune page parameters were automatically optimized injecting taurocholic acid at 1 μM as standard. The MS/MS detection was operated in MRM mode using a collision energy of 20 (arbitrary units), the observed transitions were: tauromuricholic acid (t-MCA) at 13.5 min MRM of 514.28 Th→514.28 Th, taurohyocholic acid (t-HCA) at 15.6 min MRM of 498.29 Th→498.29 Th, taurocholic acid (t-CA) at 16.6 min MRM of 514.28 Th→514.28 Th, taurochenodeoxycholic acid (t-

CDCA) at 18.5 min MRM of 498.29 Th→498.29 Th, taurodeoxycholic acid (t-DCA) at 18.9 min MRM of 498.29 Th→498.29 Th, taurolithocholic acid (t-LCA) at 22.3 min MRM of 482.29 Th→482.29 Th and tauro-10 (t-10) at 25.3 min MRM of 510.29 Th→510.63 Th.

Un-conjugated bile acids were separated at a flow rate of 200 μL/min using 10 mM ammonium acetate in water at 0.005% formic acid as the mobile phase A 10 mM ammonium acetate in methanol at 0.005% formic acid as mobile phase B. The gradient program started at 60% B and increased to 95% B in 25 min, kept at 95% B for 9 min then decreased to 60% B in 1 min and kept at 60% B for 10 min. ESI was performed in negative ion mode, the ion source temperature was set at 280 ◦C. The tune page parameters were automatically optimized injecting CA at 1 μM as standard. The MS/MS detection was operated in MRM mode using a collision energy of 15 (arbitrary units). The observed transitions were: hyocholic acid (HCA) at 8.9 min MRM of 391.29 Th→391.29 Th, cholic acid (CA) at 10.2 min MRM of 407.28 Th→407.28 Th, chenodeoxycholic acid (CDCA) at 13.8 min MRM of 391.29 Th→391.29 Th, deoxycholic acid (DCA) at 14.4 min MRM of 391.29 Th→391.29 Th, lithocholic acid (LCA) at 17.5 min MRM of 375.28 Th→375.28 Th and 10 at 20.5 min MRM of 403.63 Th→403.63Th

Determination of 7α-hydroxy-4-cholesten-3-one

Sample preparation. Stock solutions of 7alpha-hydroxy-4-cholesten-3-one were separately prepared at 5 mg/mL using MeOH as solvent. Five dilutions were obtained mixing 1 ng, 10 ng, 100 ng, 1 μg and 10 μg of 7α-hydroxy-4-cholesten-3-one in 50 μL of MeOH. Later on, 10 μL of glacial acetic acid and 10 mg of Girard T reagent (diluted in 40 μL of water) were added and kept in the dark at r.t. overnight (final volume of 100 μL).[3]

Mice serum sample aliquots of 50 μL were deproteinized with 500 μL of cold acetonitrile with 5% of NH_4OH vortexing for 60 min. After centrifugation at 16000 g for 10 min, the clear supernatant was transferred to a new vial, snap frozen and lyophilized. The sample was then re-dissolved in 50 μL of MeOH. Later on, 10 μL of glacial acetic acid and 10 mg of Girard T reagent (diluted in 40 μL of water) were added and kept in the dark at r.t. overnight (final volume of 100 μL).

Liquid chromatography and mass spectrometry analysis. For LC-MS/MS analysis, chromatographic separation was carried out on the HPLC–MS system LTQ XL ThermoScientific equipped with Accelera 600 Pump and Accelera AutoSampler system. The mixture was separated on a Jupiter C18 column from Phenomenex (150 x 2.00 mm) and the column flow rate was set at 150 μL/min. Samples were separated using a acetonitrile-metanol-aqueous gradient. Mobile phase A was water/MeOH/ACN at 50/33.3/16.7% in 0.1% TFA, mobile phase B was MeOH/ACN at 66.6/33.4% in 0.1% TFA. The gradient started at 35% B and increased to 95% B in 15 min, kept at 95% B for 10 min then decreased to 35% B in 1 min and kept at 35% B for 10 min. ESI was performed in positive ion mode, the ion source temperature was set at 280 °C. The MS/MS detection was operated using a collision energy of 40 (arbitrary units). 7α-hydroxy-4-cholesten-3-one modified by GT reagent gave a positive ion at m/z of 514.5 at 12.0 min and MS/MS analysis gave fragments at m/z of 455.4, 437.4, 427.4, 163.1, 151.1, 135.1, 123.1.

^{1}H NMR (400 MHz, CD_3OD) of compound **7**

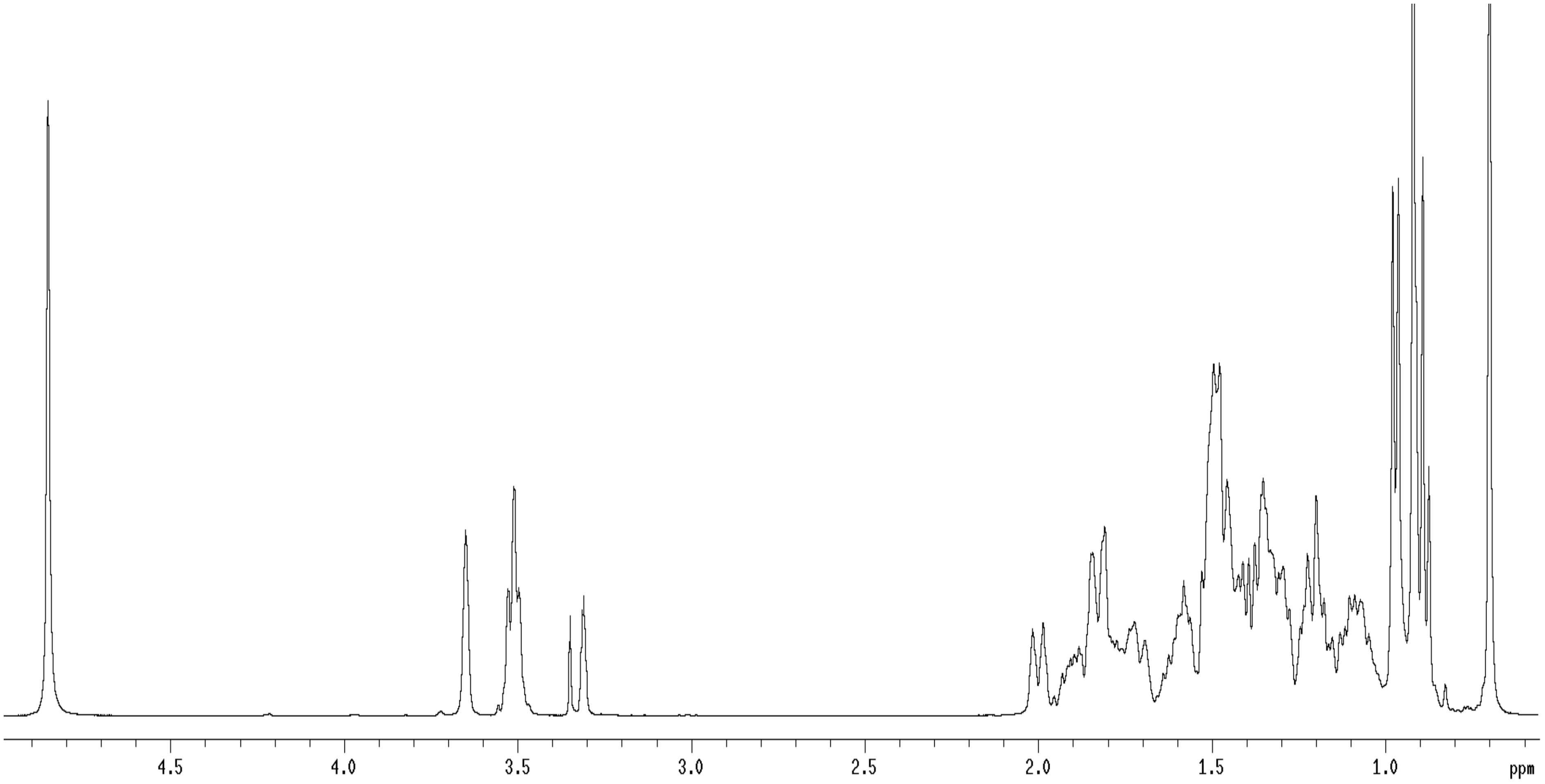

^{1}H NMR (500 MHz, CD_3OD) of compound **8**

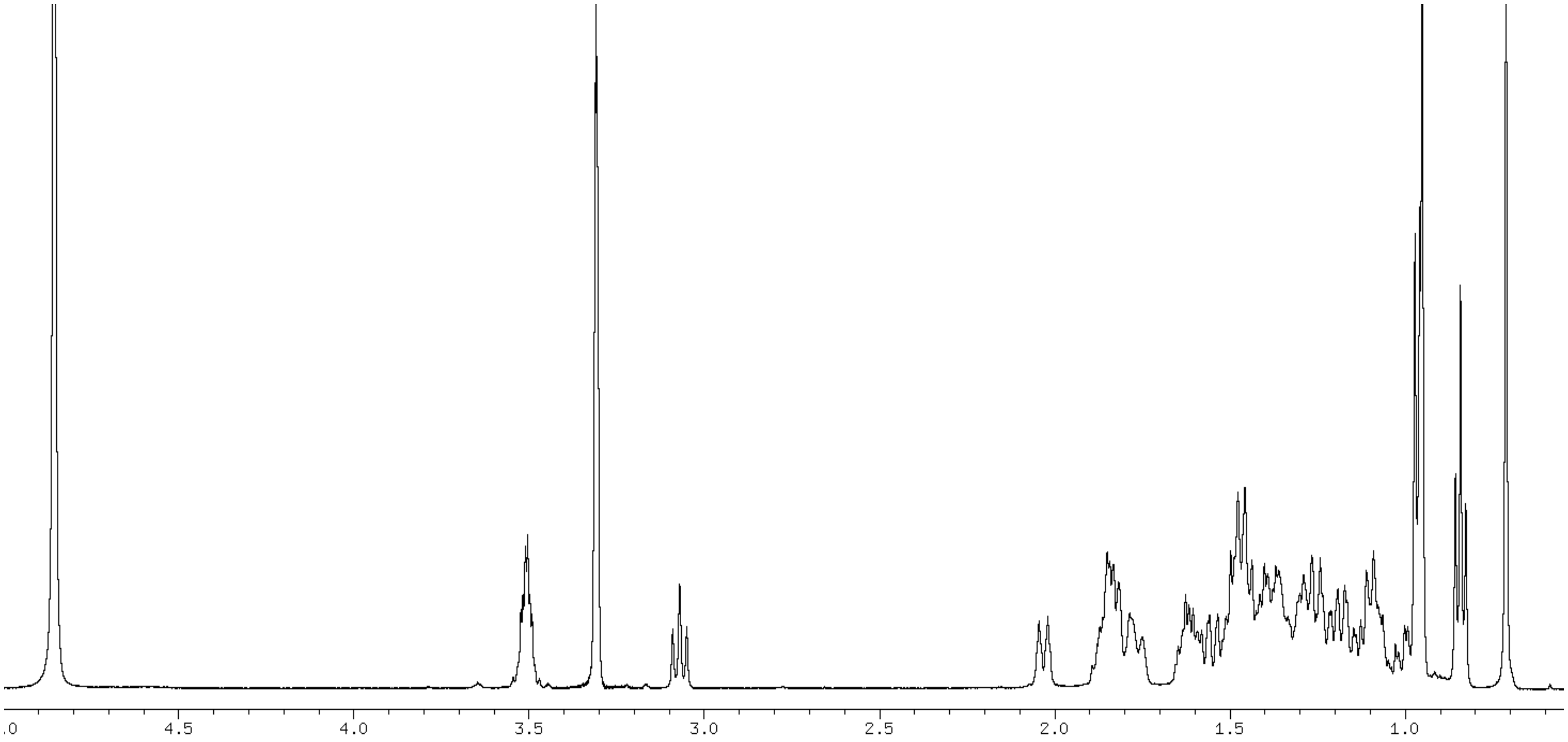

^{1}H NMR (400 MHz, CD_3OD) of compound **9**

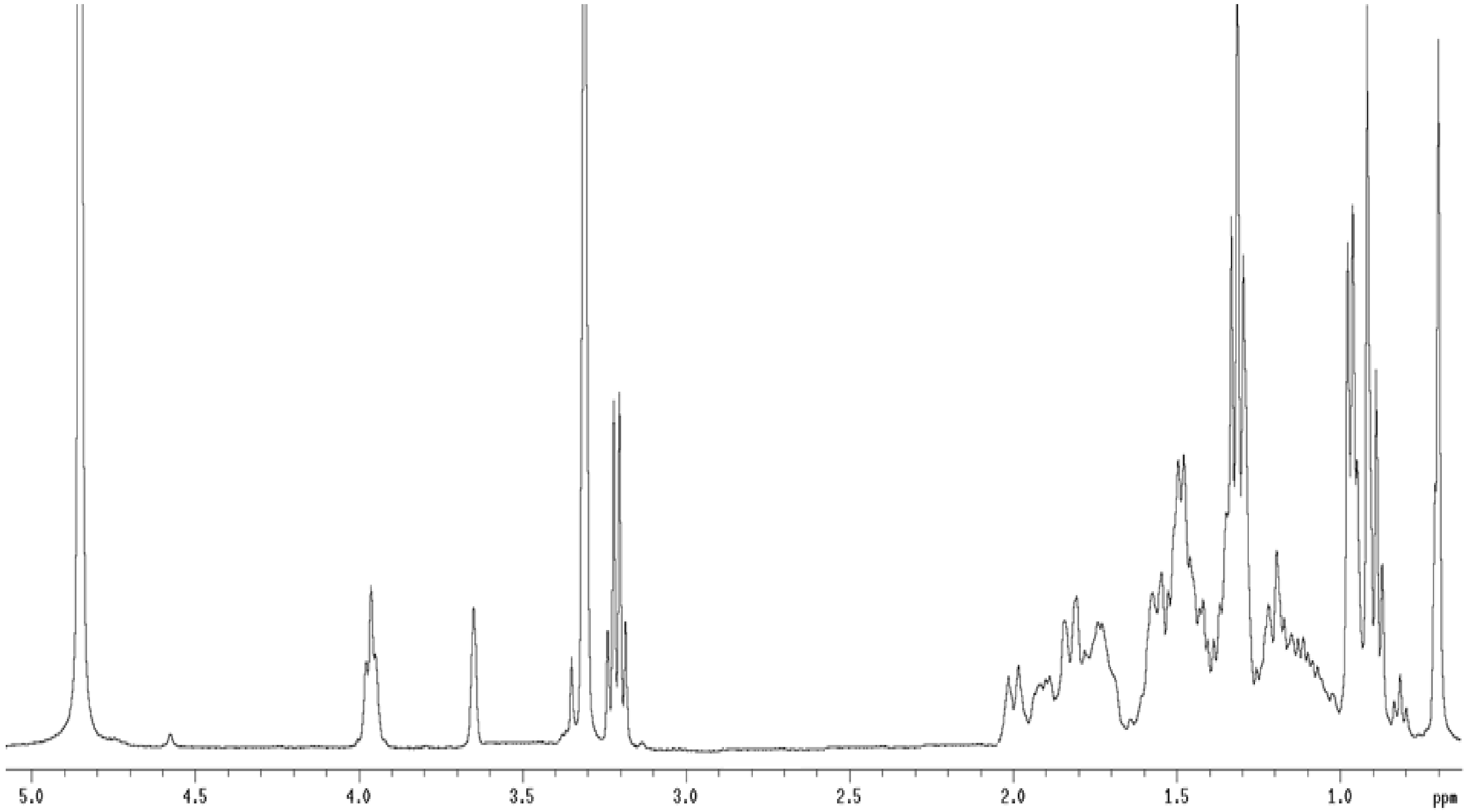

^{1}H NMR (400 MHz, CD_3OD) of compound **10**

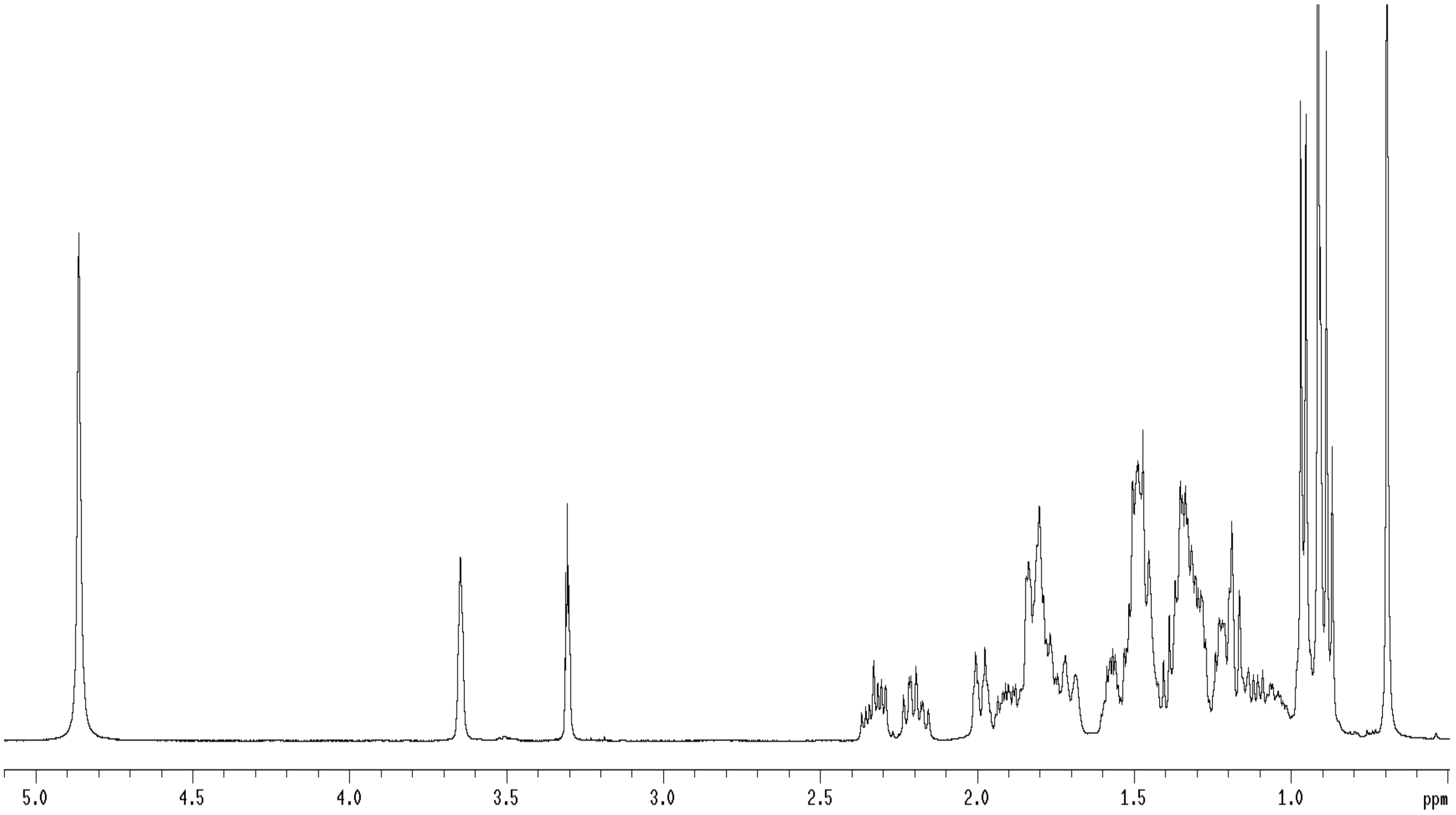

^{1}H NMR (500 MHz, CD_3OD) of compound **11**

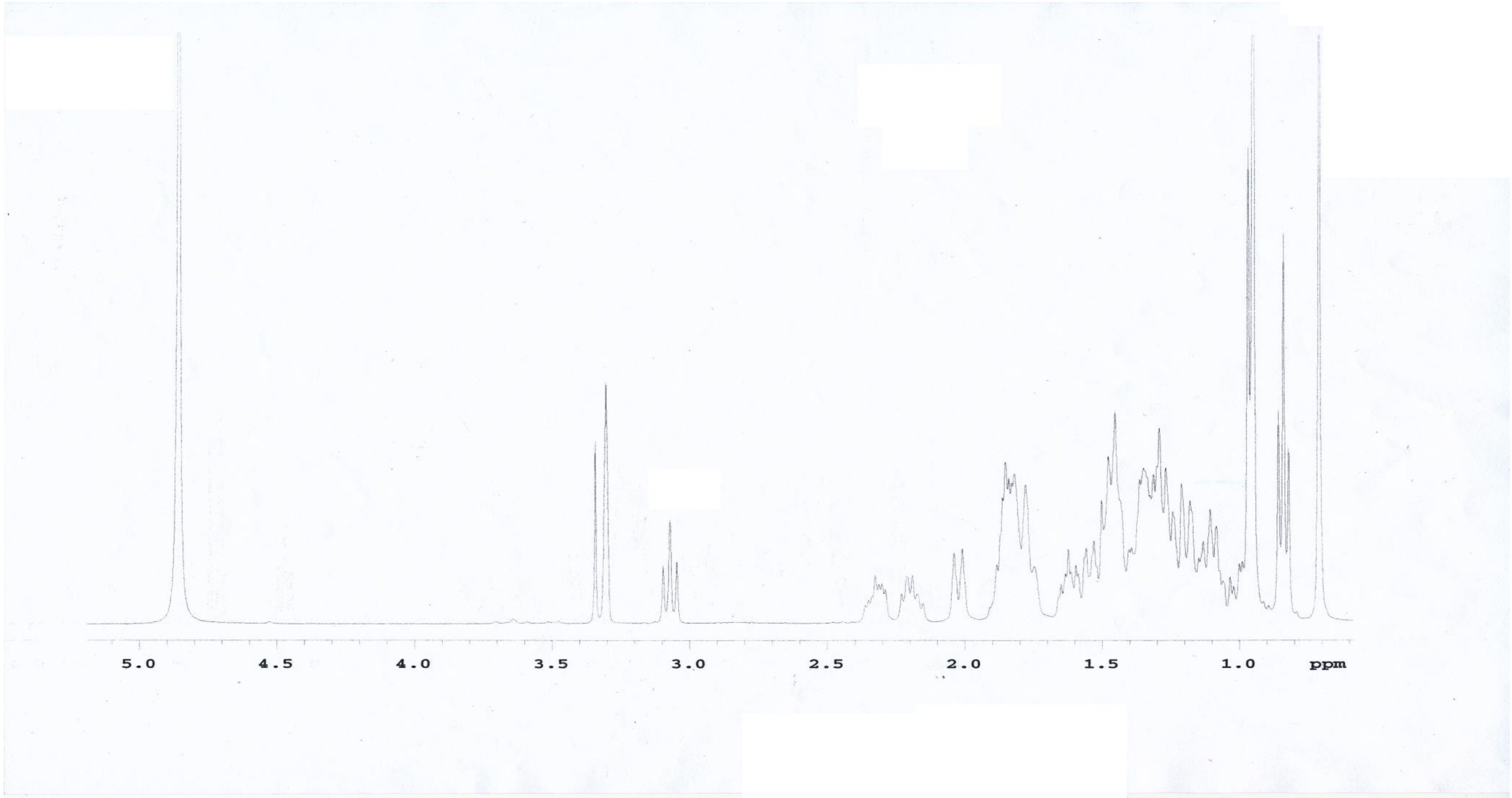

^{1}H NMR (500 MHz, CD_3OD) of compound **12**

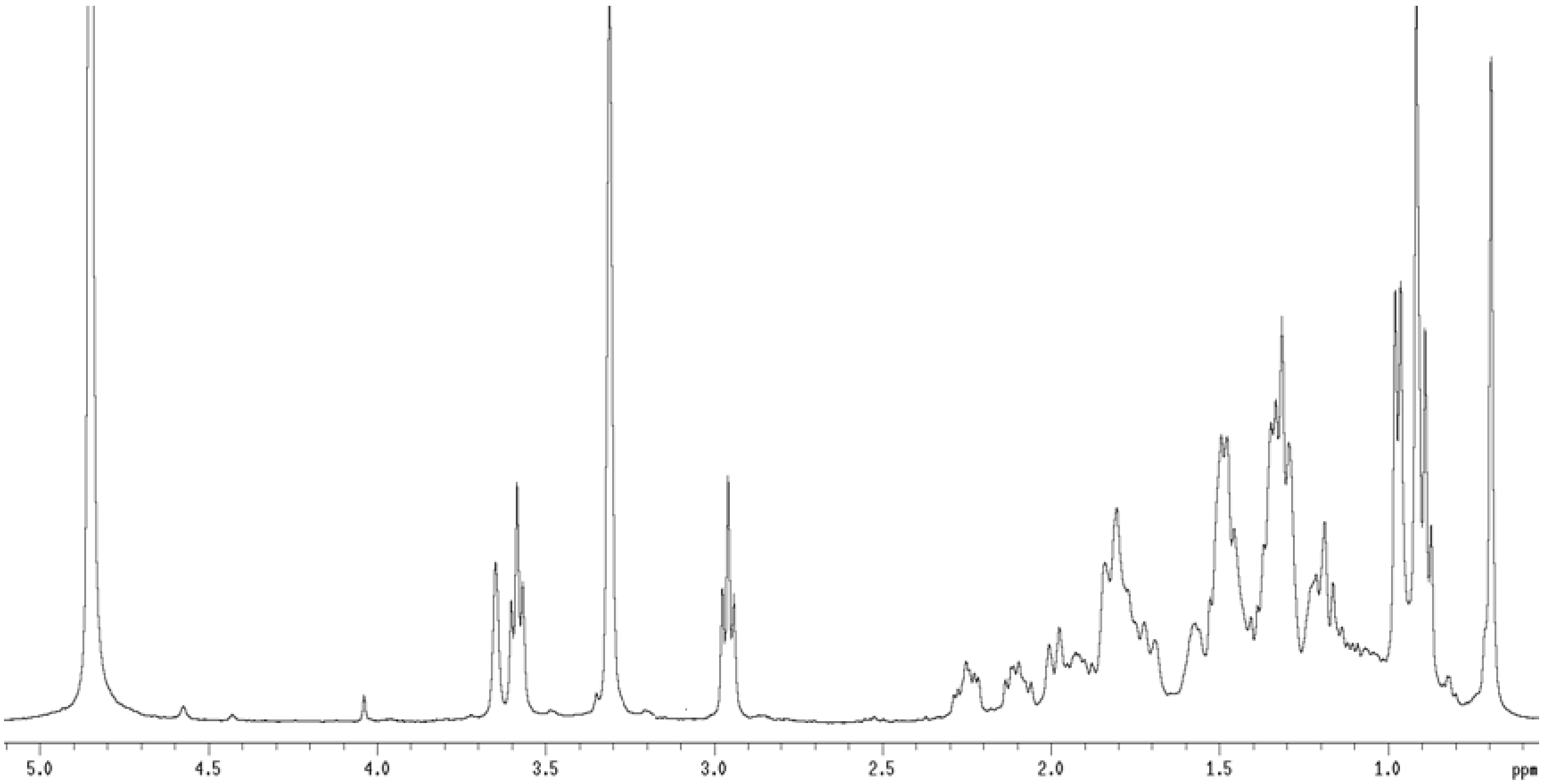

^{1}H NMR (700 MHz, CD_3OD) of compound **13**

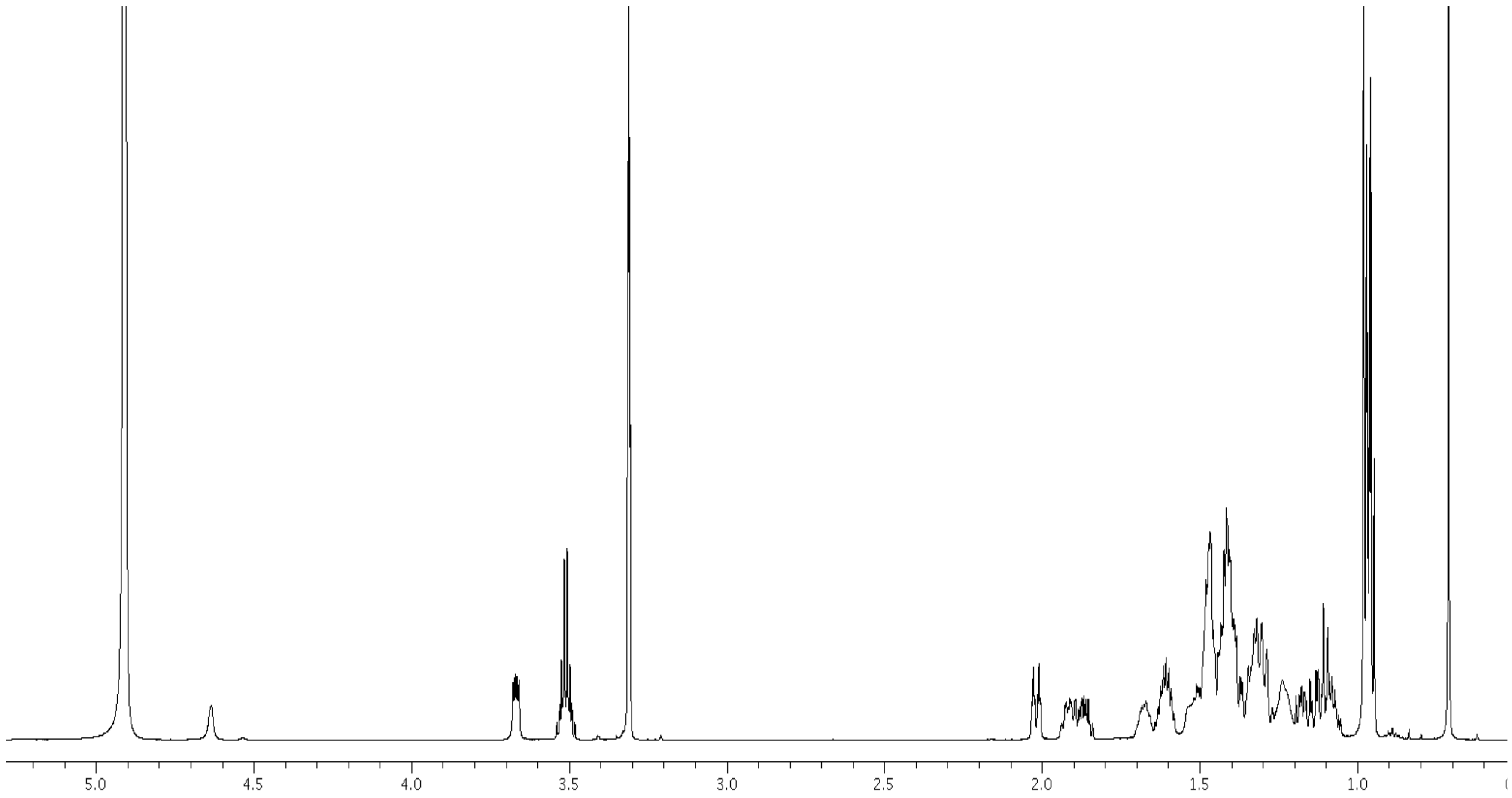

^{1}H NMR (700 MHz, CD_3OD) of compound **14**

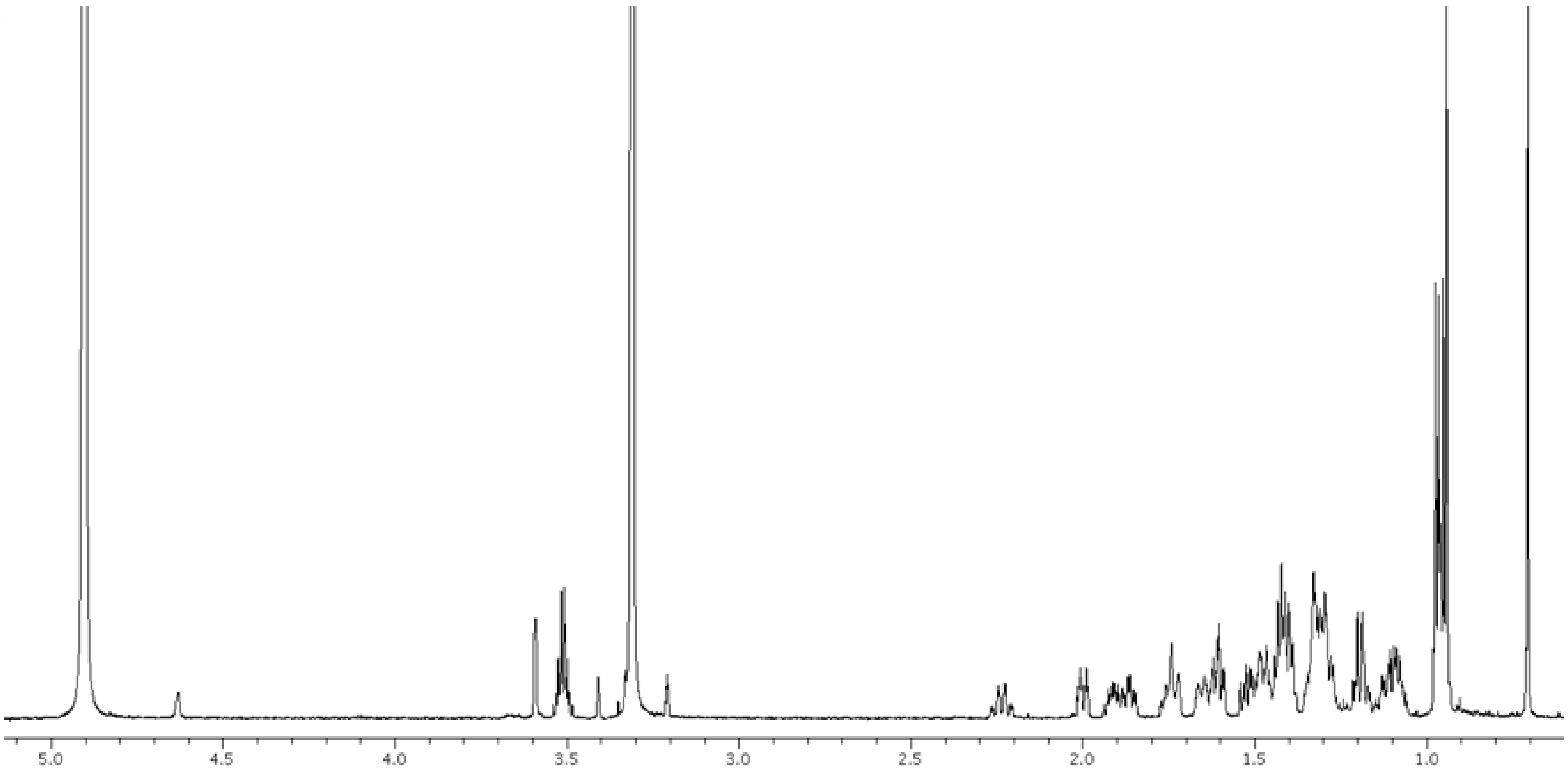

^{1}H NMR (500 MHz, CD_3OD) of compound **15**

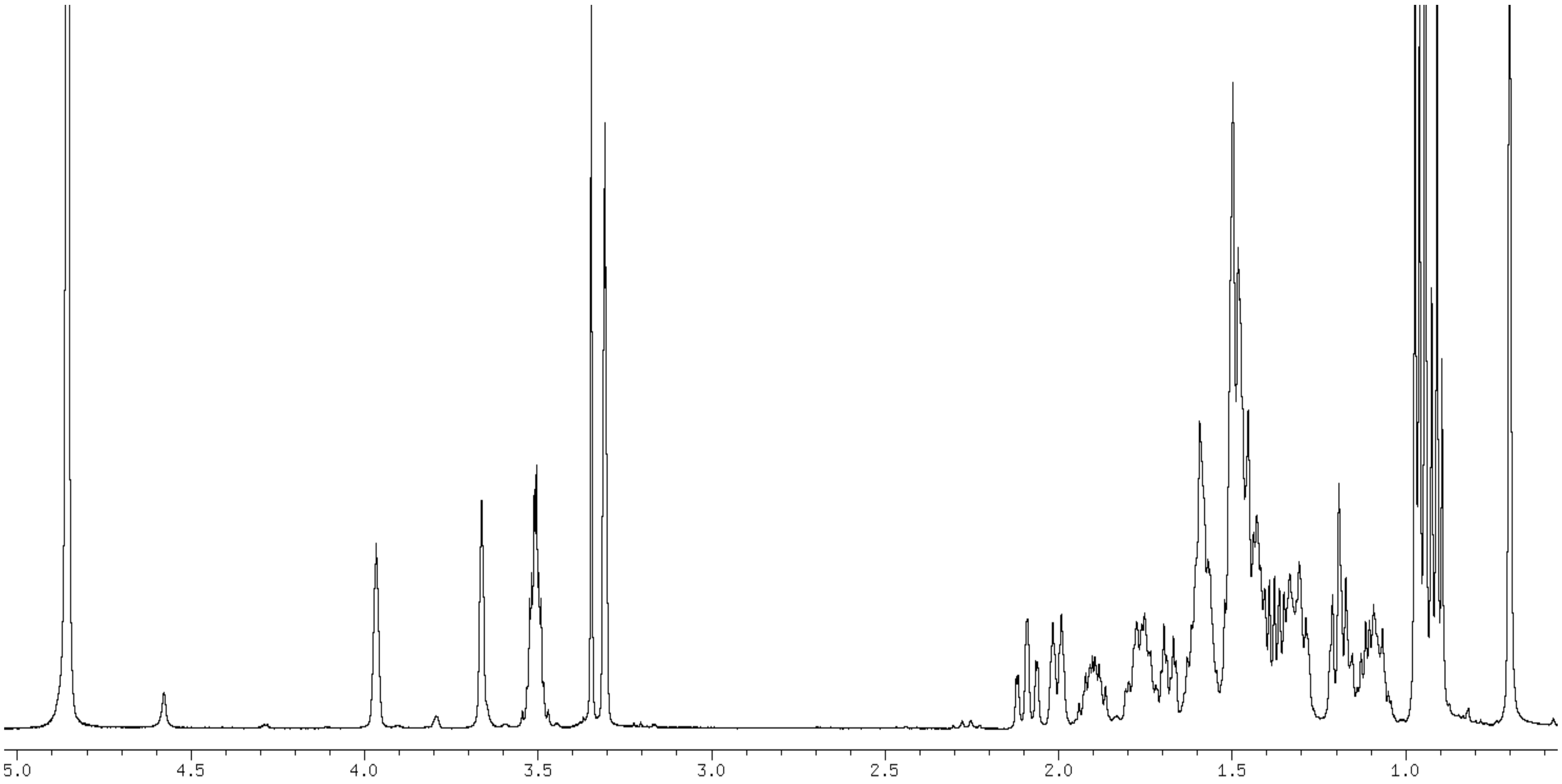

^{1}H NMR (500 MHz, CD_3OD) of compound **16**

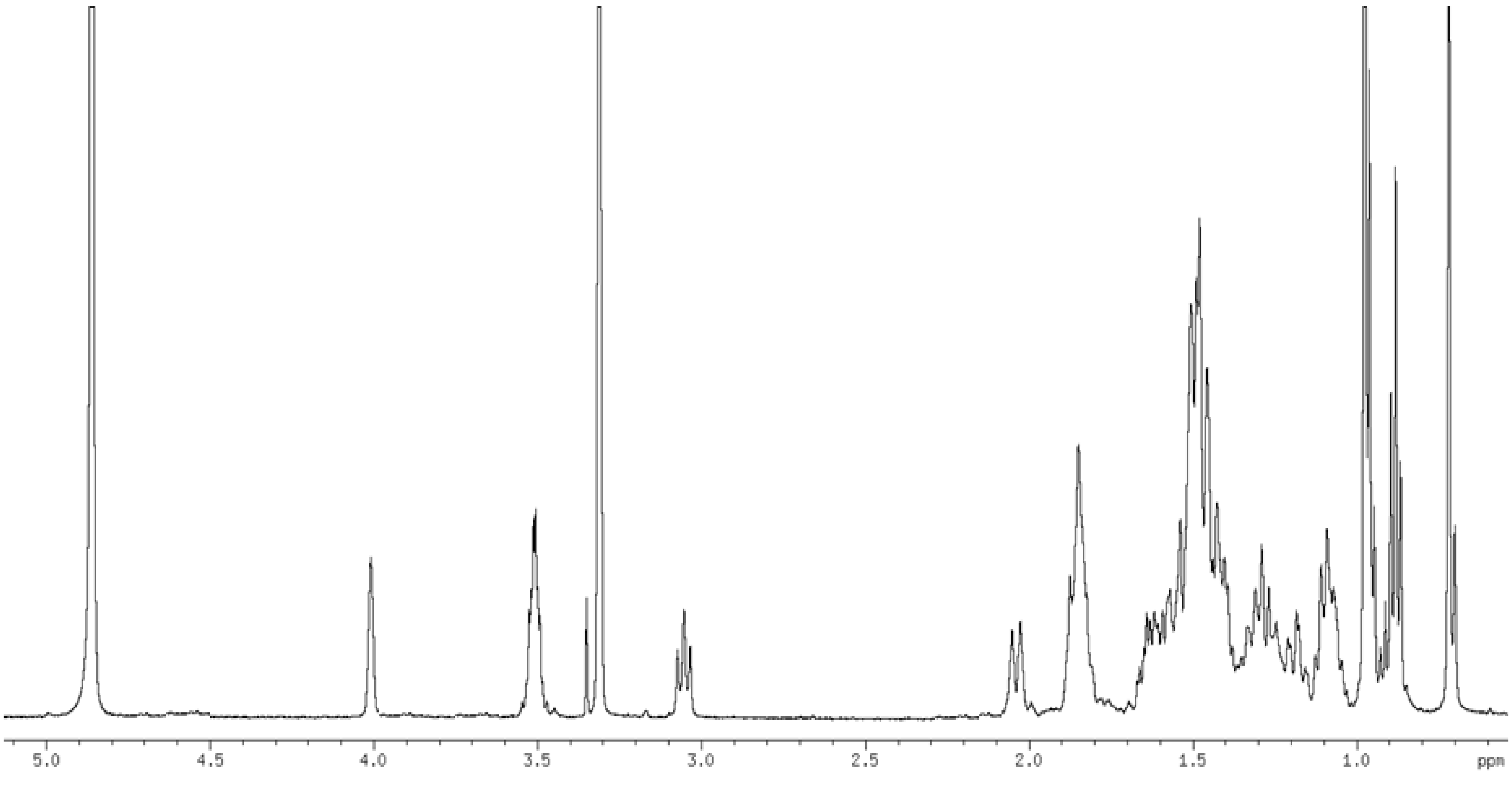

^{1}H NMR (500 MHz, CD_3OD) of compound **17**

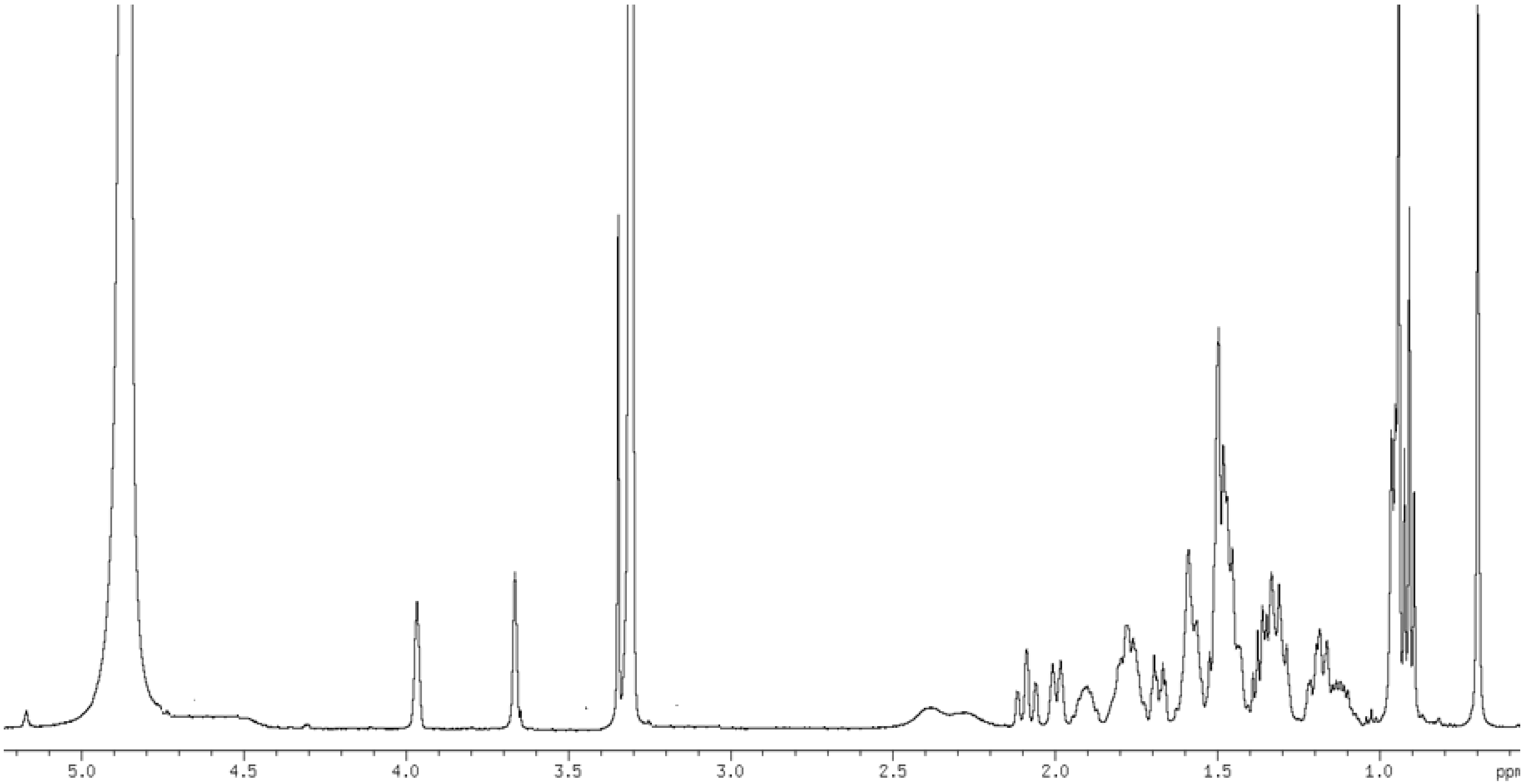

^1H NMR (500 MHz, CD_3OD) of compound **18**

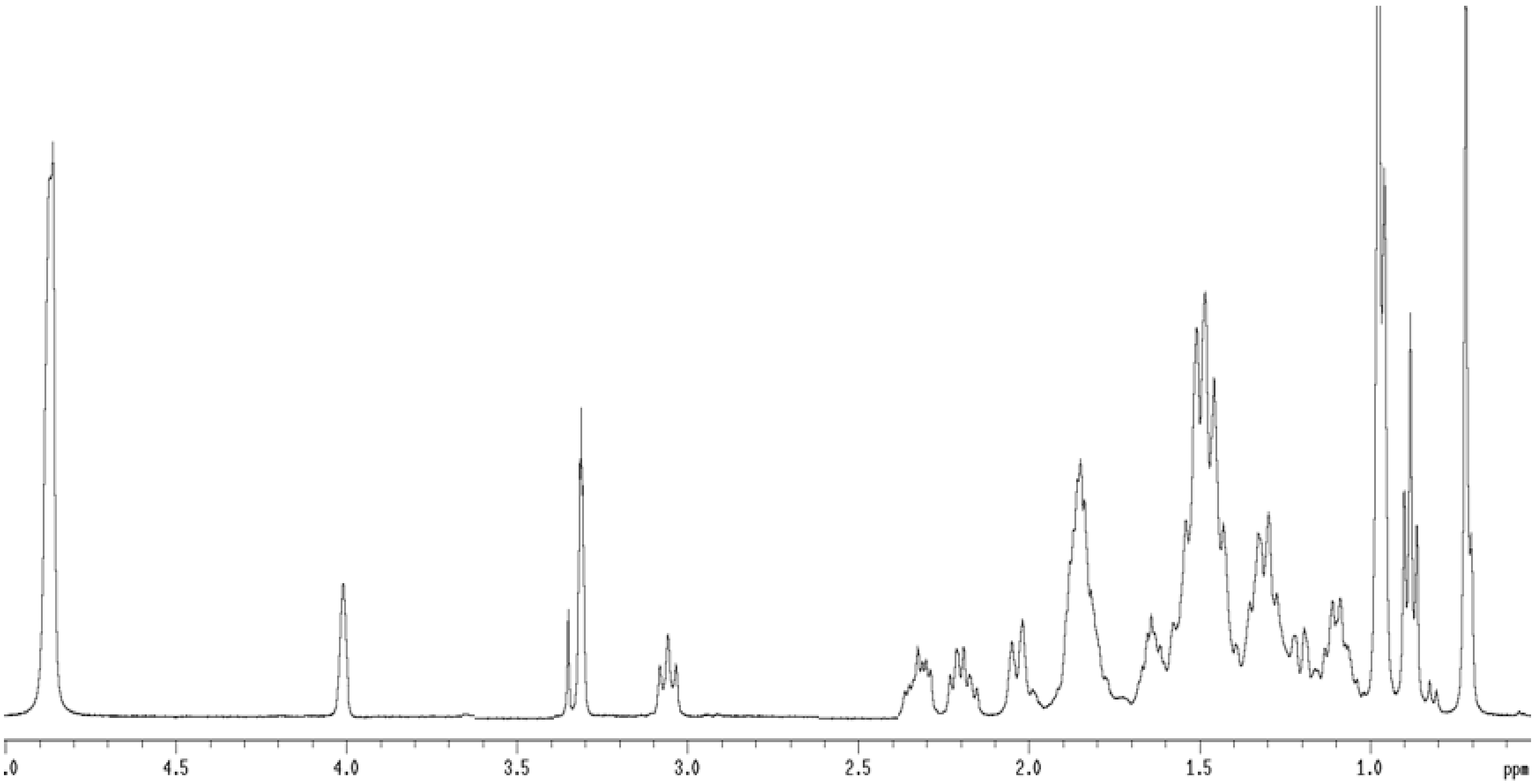

^{1}H NMR (500 MHz, CD_3OD) of compound **19**

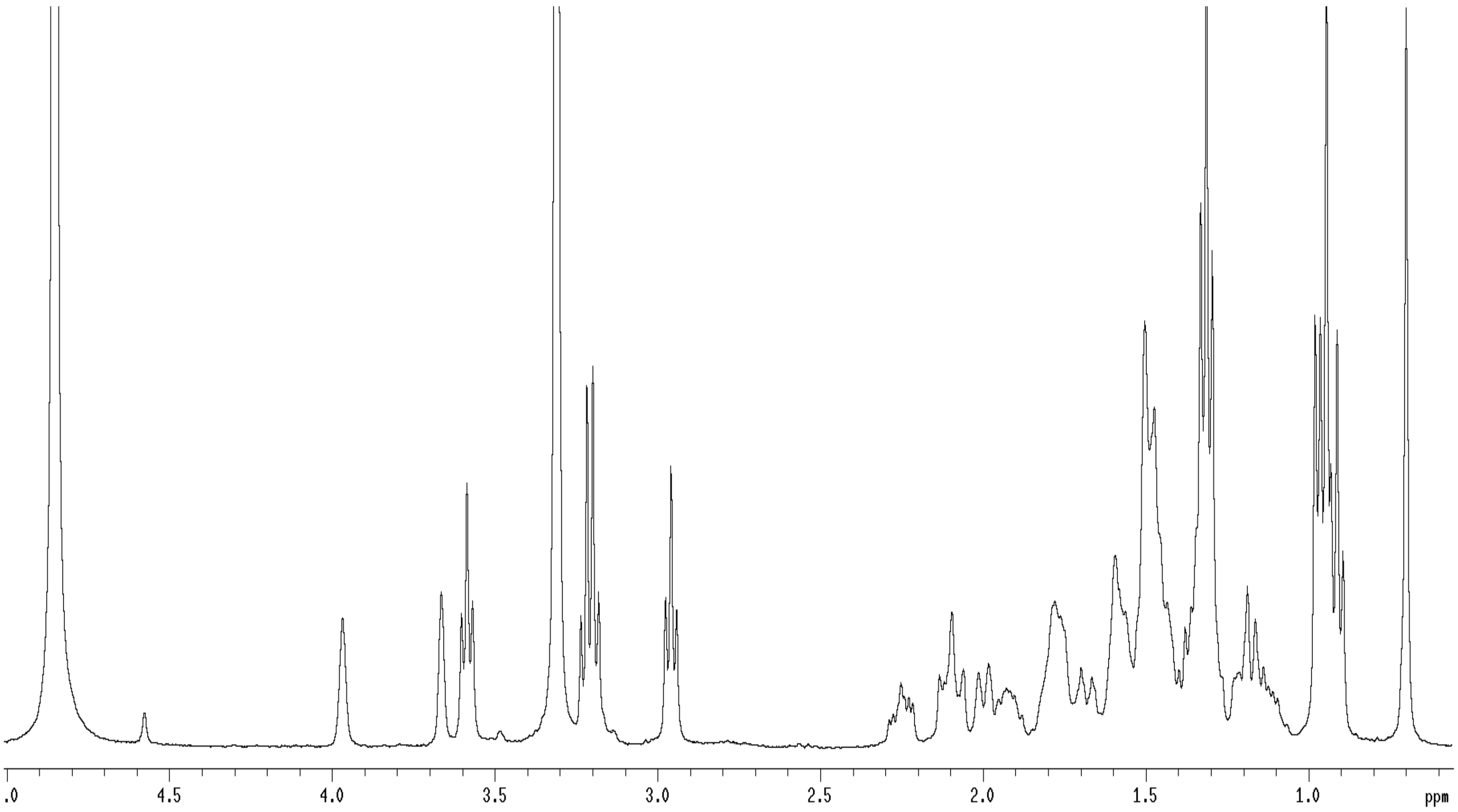

^{1}H NMR (500 MHz, CD_3OD) of compound **20**

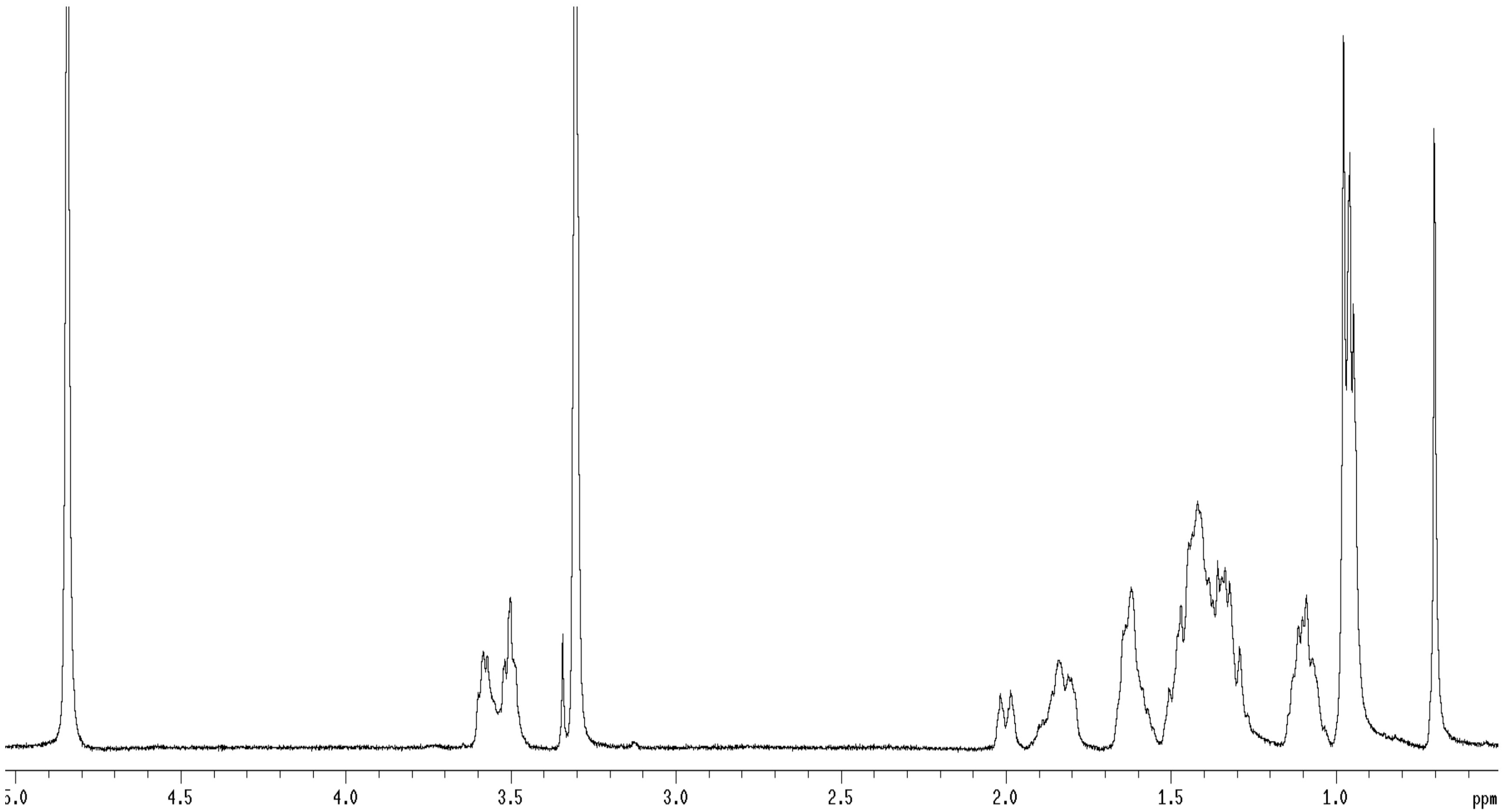

^{1}H NMR (700 MHz, CD_3OD) of compound **21**

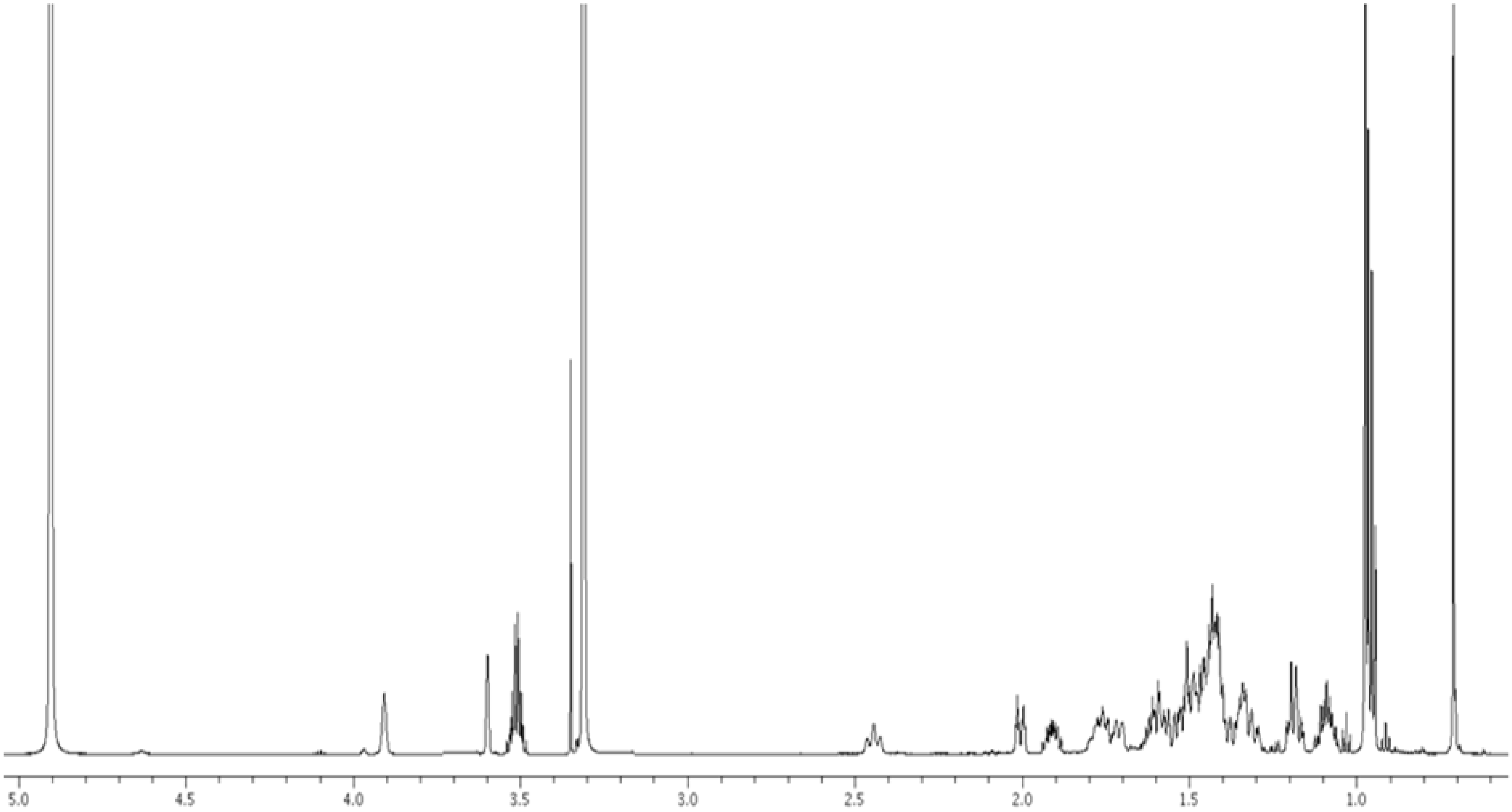

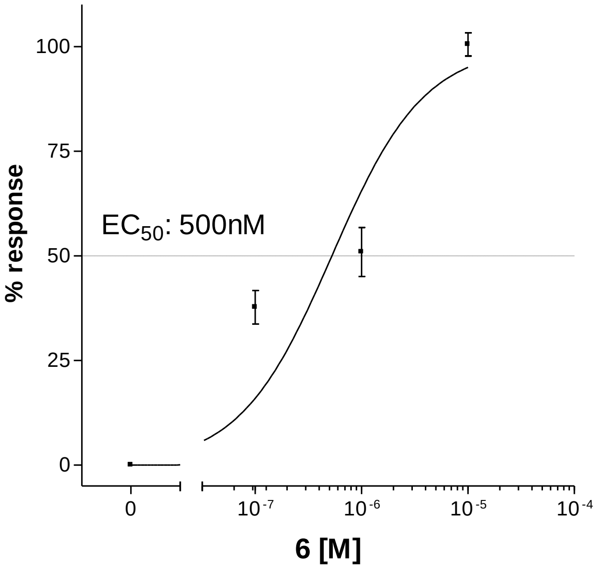

Figure S1. Concentration-response curve of 6-ECDCA (**6**) on FXR in a luciferase reporter assay using HepG2 cells transfected with FXR. Twenty-four hour post transfection cells were stimulated with increasing concentrations of **6**: range from 100 nM to 10 µM. Results are expressed as mean ± standard error.

---

1 Festa, C. *et al.* Exploitation of cholane scaffold for the discovery of potent and selective farnesoid X receptor (FXR) and G-protein coupled bile acid receptor 1 (GP-BAR1) ligands. *J. Med. Chem.* **57**, 8477-8495 (2014).

2 Sepe, V. *et al.* Modification on ursodeoxycholic acid (UDCA) scaffold. Discovery of bile acid derivatives as selective agonists of cell-surface G-protein coupled bile acid receptor 1 (GP-BAR1). J. Med. Chem, 57, 7687−7701 (2014).

3 Ogundare, M. *et al*. Cerebrospinal fluid steroidomics: are bioactive bile acids present in brain? *J. Biol Chem.* **285**, 4666-4679 (2010).
